# Origin of low melting point of ionic liquids: dominant role of entropy†

**DOI:** 10.1039/d2sc02342c

**Published:** 2022-06-08

**Authors:** Takatsugu Endo, Kouki Sunada, Hiroki Sumida, Yoshifumi Kimura

**Affiliations:** Department of Molecular Chemistry and Biochemistry, Faculty of Science and Engineering, Doshisha University 1-3 Tatara Miyakodani Kyotanabe Kyoto 610-0394 Japan taendo@mail.doshisha.ac.jp; Department of Applied Chemistry, Graduate School of Science and Engineering, Doshisha University 1-3 Tatara Miyakodani Kyotanabe Kyoto 610-0394 Japan

## Abstract

Ionic liquids (ILs) are salts with an extremely low melting point. Substantial efforts have been made to address their low melting point from the enthalpic standpoint (*i.e.* interionic interactions). However, this question is still open. In this study, we report our findings that entropic (large fusion entropy), rather than enthalpic, contributions are primarily responsible for lowering the melting point in many cases, based on a large thermodynamic dataset. We have established a computational protocol using molecular dynamics simulations to decompose fusion entropy into kinetic (translational, rotational, and intramolecular vibrational) and structural (conformational and configurational) terms and successfully applied this approach for two representatives of ILs and NaCl. It is revealed that large structural contribution, particularly configurational entropy in the liquid state, plays a deterministic role in the large fusion entropy and consequently the low melting point of the ILs.

## Abstract 2

Large structural entropy makes salts liquid at room temperature.

## Graphical abstract

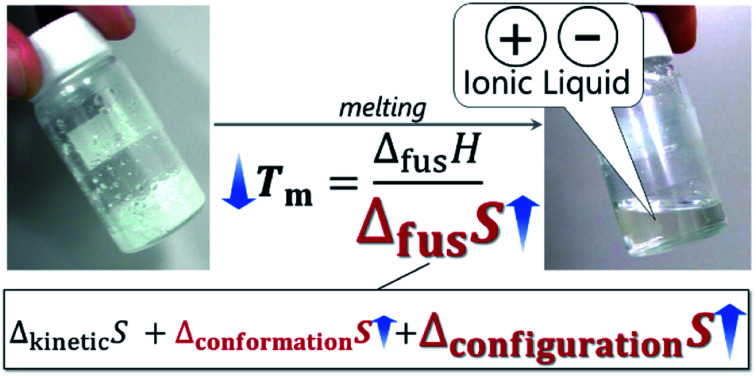

## Introduction

Salts are defined as materials composed solely of ions. Since interactions between cations and anions are governed by strong coulombic interactions, with the order of magnitudes of several hundred kJ mol^−1^, melting points of salts tend to be extremely high. The typical example is table salt (NaCl), whose melting point is 1073 K. However, in the late 1990s, scientists started to recognize salts with extremely low melting points corresponding to room temperature or even below. Nowadays, these salts are called room temperature ionic liquids or simply ionic liquids (ILs). ILs have attracted considerable attention because they have several substantial properties, such as negligible vapor pressure and flammability, high thermal/chemical/electrochemical stabilities, high ionic/electric conductivity, and unique solubilities.^1–3^ These properties mark ILs as promising candidates in a wide range of applications, for example, green solvents, electrolytes, CO_2_ absorbents, and cellulose solvents.^1–3^

The melting point is considered the most important physical property of ILs since it distinguishes them from other salts. Many investigations have been intensively conducted to answer why ILs have such a low melting point; moreover, various solutions have been proposed.^1,4–9^ Thermodynamically, contributions to the melting point are divided into two terms, *i.e.*, enthalpic and entropic terms. Although the importance of the entropic contributions (*e.g.*, conformational entropy stemming from flexible side groups) were pointed out,^1,4,6,8,9^ the mainstream discussion thus far has been made on enthalpic contributions.^1,4–9^ Namely, coulombic interaction among ions and lattice energy are weakened in ILs due to bulky, asymmetric, and sometimes charge-delocalized ions. In this work, we demonstrate that this is not the right approach and that the low melting point is caused due to entropic contribution in the majority of ILs.

## Results and discussion

### Alkali halides *versus* ionic liquids

From a thermodynamical standpoint, melting point (*T*_m_) of material is the ratio of fusion enthalpy (Δ_fus_*H*) and fusion entropy (Δ_fus_*S*):1
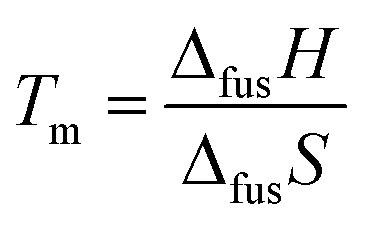


**Fig. 1:**
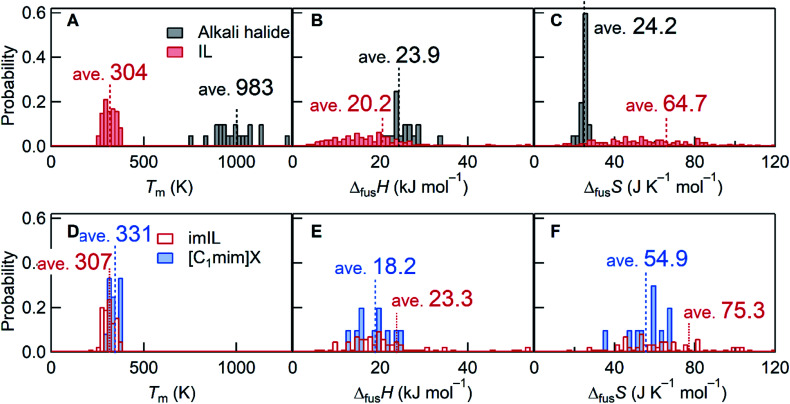
Histogram comparisons of *T*_m_, Δ_fus_*H*, and Δ_fus_*S*. (A–C) 20 alkali halides^10^*versus* 257 ILs^11,12^ and (D–F) 84 imILs *versus* 12 [C_1_mim]*X*. For [C_1_mim]*X*, this work reported 10 data and the two additional data were taken from the ref. 41 and 42 (Table S3†). Bin widths for the *T*_m_, Δ_fus_*H*, and Δ_fus_*S* histograms were 20 K, 1 kJ mol^−1^, and 2 J K^−1^ mol^−1^, respectively. For ILs and imILs, several data of Δ_fus_*H* and Δ_fus_*S* are out of range of the figures.

This rigorous expression shows that the melting point is low when Δ_fus_*H* is small and/or Δ_fus_*S* is large. Stating that the melting point of ILs is “low” requires a reference material for comparison. NaCl with a melting point of 1073 K is frequently selected. For statistical comparison purposes, 20 alkali halides were selected in this study, and their thermodynamic quantities^10^ were compared with those of 257 ILs.^11,12^ As the name suggests, the average *T*_m_ of ILs is ∼3 times lower than that of alkali halides (Fig. 1A). An average value of Δ_fus_*H* for ILs, associated with interionic interactions in the liquid and crystal states, is only 0.85 times the Δ_fus_*H* value of alkali halides (Fig. 1B). Meanwhile, the average Δ_fus_*S* of ILs is 2.67 times larger than that of alkali halides (Fig. 1C), which indicates that, in general, the large Δ_fus_*S* plays a more critical role than the small Δ_fus_*H* for lowering *T*_m_ of ILs.

Since the discussion would depend on the ions constituting the IL, we focus on imidazolium-based ILs (imILs) as the most representative IL series. Analyzing the available data on 84 imILs,^11,12^ the importance of the large Δ_fus_*S* is emphasized as seen in Fig. 1D–F. While Δ_fus_*H* values are comparable, their Δ_fus_*S* values are 3.11 times larger than those corresponding to alkali halides. Considering the origin of the large Δ_fus_*S* of ILs (or imILs), a prominent contribution may come from conformational entropy as described above.^1,4,6,8,9^ For example, most IL cations contain flexible alkyl chains that produce multiple conformations in the liquid state. This increases the entropy of the liquid state and consequently Δ_fus_*S*. An approach to thermodynamically elucidate the role of conformational entropy is to compare Δ_fus_*S* of imILs with flexible side chains to those without flexible groups. However, such data is rare. Therefore, we synthesized ten 1,3-dialkylimidazolium salts ([C_1_mim]*X*) with no conformational entropy in the cation and the anion and measured *T*_m_, Δ_fus_*H*, and Δ_fus_*S*. The difference in *T*_m_ between [C_1_mim]*X* and imILs is rather low, whereas both Δ_fus_*H* and Δ_fus_*S* of [C_1_mim]*X* are smaller than those of imILs (Fig. 1D–F). The small Δ_fus_*H* and Δ_fus_*S* values would be primarily due to the losses of inter-chain interactions^1,6–9^ and (as expected) conformational entropy, respectively. However, even without conformational entropy, not the small Δ_fus_*H* (18.2 kJ mol^−1^) but the large Δ_fus_*S* (54.9 J K^−1^ mol^−1^) seems to prevail in lowering *T*_m_ of ILs, compared to alkali halides (Δ_fus_*H* = 23.9 kJ mol^−1^ and Δ_fus_*S* = 24.2 J K^−1^ mol^−1^).

### Fusion entropy decomposition

**Fig. 2:**
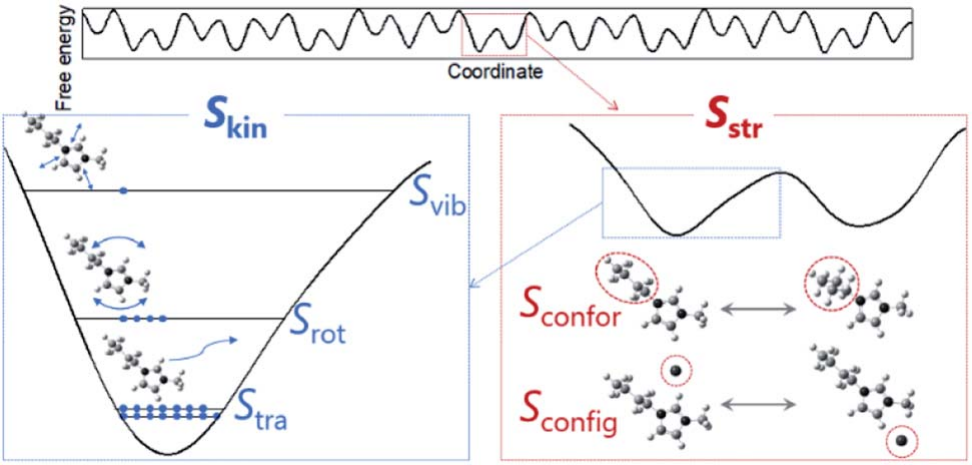
Schematic of entropies based on the free energy landscape. Absolute entropy is expressed as the sum of kinetic (*S*_kin_) and structural (*S*_str_) contributions. Kinetic entropy corresponds to the number of distinguishable states where a particle of interest occupies (blue symbols) in a potential well. Structural entropy, corresponding to the number of potential wells, is expressed as the sum of conformational (or intramolecular) (*S*_confor_) and configurational (or intermolecular) (*S*_config_) parts. *S*_config_ is displayed based on an ion-pair model where an anion is drawn as a monoatomic ion.

To quantitatively unravel ambiguous entropic contributions, entropy needs to be decomposed into individual components associated with molecular-level properties. The decompositions were conducted based on the free energy landscape model^13,14^ (Fig. 2). Absolute entropy (*S*) can be expressed as the sum of kinetic (*S*_kin_) and structural (*S*_str_) entropies. The former is further divided into translational (*S*_tra_), rotational (*S*_rot_), and intramolecular vibrational (*S*_vib_) terms and the latter is composed of conformational/intramolecular (*S*_confor_) and configurational/intermolecular (*S*_config_) entropies. Since we focus on the difference in entropy between liquids and crystals at the melting point, by assuming individual terms are independent, appropriate equations are as follows:

2 Δ_fus_*S* = Δ_kin_*S* + Δ_str_*S*

3 Δ_kin_*S* = Δ_tra_*S* + Δ_rot_*S* + Δ_vib_*S*

4 Δ_str_*S* = Δ_confor_*S* + Δ_config_*S*

To perform the Δ_fus_*S* decomposition, we have developed a computational protocol using classical molecular dynamics (MD) simulations (see the ESI† for details). Δ_fus_*S* and Δ_confor_*S* were estimated from the thermodynamic integration^15,16^ and conformational analyses, respectively. We employed the two-phase thermodynamic (2PT) approach, initially developed by Lin *et al.*, for the kinetic entropy estimations.^17–19^ Conventionally, the entropy of vibrational motions for molecules in a solid state has been estimated through the density of states functions *g*(*v*) by assuming that all vibrational motions are harmonic oscillators. However, this technique cannot treat diffusive motions existing in a liquid state. The 2PT approach enables the estimation of entropy in a liquid state by dividing *g*(*v*) in the liquid state into solid-like (for harmonic oscillators) and gas-like (for diffusive motions) components. The remaining Δ_config_*S* was obtained based on eqn (2)–(4) by the deduction of the other terms. For reliable discussion on results obtained from classical MD simulations, a selection of force fields is critical. We selected the force fields developed by Maginn *et al.* for two prototype imILs as 1-ethyl-3-methylimidazolium hexafluorophosphate ([C_2_mim]PF_6_) and 1-butyl-3-methylimidazolium hexafluorophosphate ([C_4_mim]PF_6_).^20^ Partial charges in these force fields were determined by *ab initio* MD in the crystal state with periodic boundary conditions so that the charge transfer and polarization in the condensed state are implicitly included. Furthermore, MD simulations on NaCl with the Tosi–Fumi potential^21^ were conducted for comparison. Simulated *T*_m_, Δ_fus_*H*, and Δ_fus_*S* were in good agreement with the experimental values (Table S7†). It is noted that the absolute entropies also satisfactorily reproduced experimental data (Table S10†).

**Fig. 3:**
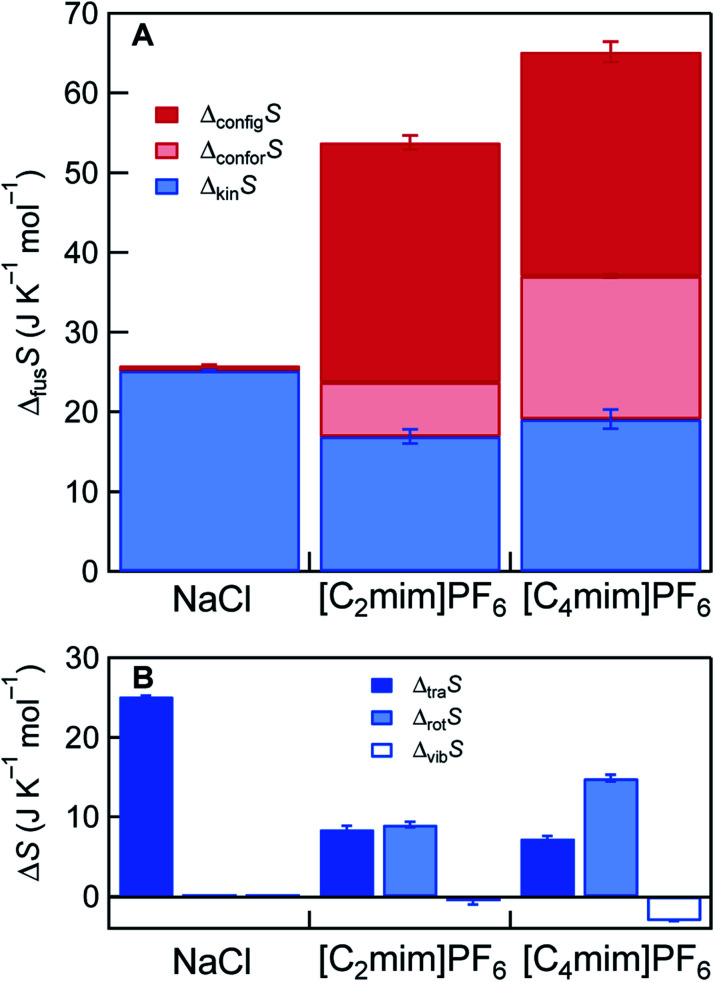
Decomposition of simulated Δ_fus_*S*. For [C_2_mim]PF_6_ and [C_4_mim]PF_6_, Δ_vib_*S* shows slight negative values, hence Δ_kin_*S* (=Δ_tra_*S* + Δ_rot_*S* + Δ_vib_*S*) is displayed in (A). Individual Δ_tra_*S*, Δ_rot_*S*, and Δ_vib_*S* are shown in (B).

The results of the Δ_fus_*S* decompositions for NaCl and the two ILs are presented in Fig. 3. In NaCl that is composed of monoatomic ions, the following stands: Δ_fus_*S* ≈ Δ_kin_*S* = Δ_tra_*S*. For the ILs, Δ_kin_*S* is smaller than that of NaCl, even though the ILs have larger absolute entropy than NaCl in any state of matter, mainly due to the presence of *S*_rot_ and *S*_vib_ (Fig. S14†). There are three reasons for this counter-intuitive behavior. First, Δ_vib_*S* of the ILs does not contribute to Δ_kin_*S*, or they are even slightly negative. This would result from the fact that intramolecular vibrations are mostly unchanged by melting, as suggested by previous reports.^22,23^ The second reason is related to the difference in melting point. Since the melting points of the ILs are intrinsically lower than that of NaCl, diffusive motions (translational + rotational) represented by *g*(0) (density of states at zero frequency) are not activated by melting in the ILs, compared to NaCl (Fig. S11–S13 and Table S12†). The slow diffusive motions are also expected for other ILs since their viscosity is generally as high as several tens or more mPa s at the melting point. Third, the spherical PF_6_^−^ anions already gain rotational diffusivity in the crystal state (Fig. S12C and S13C†), consistent with previous NMR observations,^24^ resulting in lowering Δ_rot_*S*.

Despite the small Δ_kin_*S* of the ILs, the presence of large Δ_str_*S* (=Δ_confor_*S* + Δ_config_*S*) is the cause for the large Δ_fus_*S*. The presence of Δ_confor_*S* has been previously pointed out by several groups.^1,4,6,8,9^ The calculated Δ_confor_*S* values were 6.8 J K^−1^ mol^−1^ for [C_2_mim]PF_6_ and 18.0 J K^−1^ mol^−1^ for [C_4_mim]PF_6_. These values are slightly smaller than the previously reported *S*_confor_ in the gas state (8.3 J K^−1^ mol^−1^ for [C_2_mim]PF_6_ and 22.8 J K^−1^ mol^−1^ for [C_4_mim]PF_6_).^25^ This would result from the fact that *S*_confor_ in the crystalline state was calculated to be non-zero due to the slight disordering of the alkyl group in the cation (Tables S8 and S9†). A significant feature in Fig. 3A is that Δ_config_*S* plays a more prominent role in lowering *T*_m_ than Δ_confor_*S*. The Δ_config_*S* (=here *S*_config_ in the liquid state) values of [C_2_mim]PF_6_ and [C_4_mim]PF_6_ were estimated to be 30.0 J K^−1^ mol^−1^ and 28.1 J K^−1^ mol^−1^, respectively. Assuming *S*_config_ = *R* ln *W*, where *W* is the number of distinguishable microscopic states, at least 30–40 potential energy basins exist for these ILs in the liquid state. Although multiple configurations in ILs were already pointed out, and they were associated with the low melting point in a few papers,^8,26^ we quantitatively demonstrate the dominant role of Δ_config_*S* in the low melting point of ILs. The origin of multiple configurations is considered to come from the delocalized charges in the ion and the asymmetric ion structure.^26^ It is worth noting that these characteristics of ILs have been previously considered to contribute to lowering the melting point enthalpically by reducing coulombic interactions and packing efficiency.^1,6,8,9^ Our findings indicate that the delocalized charge and the asymmetric ion structure can be entropically, rather than enthalpically, important for the low melting point. Since large Δ_kin_*S* cannot be expected for general ILs, because of close-to-zero Δ_vib_*S* and high viscosity (small Δ_tra_*S* + Δ_rot_*S*), large Δ_str_*S* (particularly large Δ_config_*S*) is the key for the large Δ_fus_*S* and the consequent low melting point of ILs.

## Conclusions

In this work, we have discovered that the low melting point of ILs is mainly driven entropically, *i.e.*, the large Δ_fus_*S* dominantly decreases the melting point of ILs. To unravel the origin of the large Δ_fus_*S*, imILs as the most representative IL series were focused. With the developed protocol based on MD simulations, Δ_fus_*S* of the two imILs as well as NaCl were successfully decomposed into individual components with a clear physical origin. The simulations indicated that, somewhat counterintuitively, not the kinetic but the structural part, particularly Δ_config_*S*, was the main contributor to the low melting point of the ILs. Breaking the mainstream of the previous “enthalpic” discussion, our findings based on the “entropic” contributions can change the bottom concept for designing ILs and could consequently accelerate the production of a number of novel functional ILs.

## Experimental

### Selection of thermodynamic data of ILs

The thermodynamic data, *T*_m_, Δ_fus_*H*, Δ_fus_*S* for ILs were taken from the ILThermo database as summarized in Table S1.†^11,12^ (It is noted that *T*_m_, Δ_fus_*H*, Δ_fus_*S* for alkali halide are also summarized in Table S2†).^10^ The ILThermo database seems to contain some inappropriate data for discussion, *i.e.*, *T*_m_ is too high to be ILs, or Δ_fus_*H* or Δ_fus_*S* is too low to be considered as enthalpy/entropy difference on melting. In this work, we excluded the data with *T*_m_ > 373.2 K, Δ_fus_*H* < 3 kJ mol^−1^, or Δ_fus_*S* < 10 J K^−1^ mol^−1^ from the discussion. In some cases, several different data were reported in one IL because of, *e.g.*, differences in experimental conditions, experimental error, and purities as well as the existence of polymorphism. Considering the purity and thermodynamic stability of polymorphs, we selected the one with the highest melting point.

### IL syntheses and characterizations

The ILs [C_1_mim]*X* were synthesized following standard procedures.^1^ The detailed procedures were described in the ESI.† The ILs were vacuum-dried with heating for several days (typically 353 K for 2 days) before use. The synthesized ILs were characterized with NMR spectroscopy (JEOL, JNM-ECA300W). No recognizable impurities were observed *via* NMR spectra except for water. The water content of the ILs was determined by Karl Fischer titration (MKC-501, Kyoto Electronics). When an IL was in the crystal state at room temperature, the water content measurement was conducted by dissolving it in anhydrous acetonitrile. Several ILs potentially contain a slight amount of alkali halides as a byproduct, the content of which was determined by Na^+^ meter (LAQUAtwin Na-11, Horiba) or the Mohr's method.

### DSC measurements

Differential scanning calorimetry (DSC) measurements were performed with a DSC7020 (Hitachi High-Tech Science). A dried IL was sealed in an Al pan in an Ar-atmosphere glovebox. In the measurements, the sample was first melted by heating, and data was subsequently collected by cooling down to 175 K and re-heating above the melting point at a scanning rate of 5 K min^−1^. The obtained DSC traces are shown in Fig. S1,† and the numerical values are listed in Tables S3 and S4.†

### Quantum chemical calculations

Quantum chemical calculations were performed to estimate absolute entropies of NaCl, [C_2_mim]PF_6_, and [C_4_mim]PF_6_ in the gas phase with Gaussian09^27^ program package. B3LYP^28–30^ combined with GD3 (ref. 31) correction at 6-311++G(d,p) level were employed for the calculations. Full geometry optimization and subsequent vibrational frequency analyses were performed. The initial structure of the IL ion pairs for the geometry optimization was referred to the ones reported previously^32^ (Fig. S2†). It is noted that no imaginary frequency was obtained for the optimized structures, which confirms that they are local minima. The absolute entropies and the decomposed kinetic entropies (*S*_tra_, *S*_rot_, and *S*_vib_) for NaCl, [C_2_mim]PF_6_, and [C_4_mim]PF_6_ are summarized in Table S5.†

### Molecular dynamics simulations

Classical MD simulations were performed with Gromacs 2018.4 Software package^33,34^ (single precision) with the periodic boundary condition. Newton's equation of motion was solved in 1 fs time step with the leap-frog algorithm unless otherwise stated. Cut-off radii for both coulombic and LJ potentials were set to be 1.2 nm. Long-range dispersion corrections were applied. Long-range coulombic interactions were treated with the particle mesh Ewald summation method.^35,36^ Temperature and pressure were controlled with Nose–Hoover thermostat and Parrinello–Rahman barostat, respectively. No constraint was applied for intramolecular bonds and angles.

Ion pairs of 2048 (NaCl), 400 ([C_2_mim]PF_6_), and 250 ([C_4_mim]PF_6_) were used in MD simulations. The force field of NaCl was based on the Tosi–Fumi potential.^21^ The ones developed by Maginn *et al.*^20^ with the aid of the general AMBER force field^37,38^ were used for the two ILs. A crystal structure with fcc lattice was employed for the NaCl crystal. Crystal structures of [C_2_mim]PF_6_ (ref. 39) and [C_4_mim]PF_6_ (ref. 40) were taken from the references. For liquid state simulations, ions were randomly placed into a cubic cell. After energy minimizations with the steepest descent algorithm, an initial simulation box was first equilibrated in the NPT ensemble until system energy became constant, at least for 2 ns. If necessary, a subsequent NVT ensemble simulation was conducted with the equilibrated cell size. For statistical error estimation, a production run after the equilibration was equally divided into 4 blocks. The standard deviation based on 4 simulations was taken as the statistical error. It is noted that in the figures displayed in the main text and the ESI,† the average data of the 4 blocks are shown. Detailed procedures of estimations of *T*_m_, Δ_fus_*H*, Δ_fus_*S*, and the entropies were described in the ESI.†

## Data availability

All available data are included in the ESI.†

## Author contributions

T. Endo conceived and designed this study. K. Sunada synthesized the compounds. T. Endo performed the molecular dynamics simulations. H. Sumida performed the quantum chemical calculations. T. Endo analyzed the data. T. Endo produced the manuscript. K. Sunada, H. Sumida and K. Kimura checked and modified the manuscript.

## Conflicts of interest

There are no conflicts to declare.

## Supplementary Material

SC-013-D2SC02342C-s001

## References

[cit1] MacFarlane D. R. , Kar M. and Pringle J. M., Fundamentals of Ionic Liquids: From Chemistry to Applications, Wiley-VCH, 2017

[cit2] Rogers Robin D., Seddon Kenneth R. (2003). Science.

[cit3] Plechkova N. V., Seddon K. R. (2008). Chem. Soc. Rev..

[cit4] Krossing I., Slattery J. M., Daguenet C., Dyson P. J., Oleinikova A., Weingärtner H. (2006). J. Am. Chem. Soc..

[cit5] Zahn S., Uhlig F., Thar J., Spickermann C., Kirchner B. (2008). Angew. Chem., Int. Ed..

[cit6] Dean P. M., Pringle J. M., MacFarlane D. R. (2010). Phys. Chem. Chem. Phys..

[cit7] Nelyubina Y. V., Shaplov A. S., Lozinskaya E. I., Buzin M. I., Vygodskii Y. S. (2016). J. Am. Chem. Soc..

[cit8] Izgorodina E. I., Seeger Z. L., Scarborough D. L. A., Tan S. Y. S. (2017). Chem. Rev..

[cit9] Philippi F., Welton T. (2021). Phys. Chem. Chem. Phys..

[cit10] Janz G. J. , Molten Salts Handbook, Academic, 1967

[cit11] Dong Q., Muzny C. D., Kazakov A., Diky V., Magee J. W., Widegren J. A., Chirico R. D., Marsh K. N., Frenkel M. (2007). J. Chem. Eng. Data.

[cit12] Kazakov A. , Magee J. W., Chirico R. D., Paulechka E., Diky V., Muzny C. D., Kroenlein K. and Frenkel M., NIST Standard Reference Database 147: NIST Ionic Liquids Database – (ILThermo), Version 2.0, National Institute of Standards and Technology, Gaithersburg MD, 2021, p. 20899, http://ilthermo.boulder.nist.gov

[cit13] Berthier L., Ozawa M., Scalliet C. (2019). J. Chem. Phys..

[cit14] Stillinger F. H. (1995). Science.

[cit15] Eike D. M., Brennecke J. F., Maginn E. J. (2005). J. Chem. Phys..

[cit16] Bernardino K., Zhang Y., Ribeiro M. C. C., Maginn E. J. (2020). J. Chem. Phys..

[cit17] Lin S. T., Blanco M., Goddard III W. A. (2003). J. Chem. Phys..

[cit18] Caro M. A., Laurila T., Lopez-Acevedo O. (2016). J. Chem. Phys..

[cit19] Lin S. T., Maiti P. K., Goddard III W. A. (2010). J. Phys. Chem. B.

[cit20] Zhang Y., Maginn E. J. (2012). Phys. Chem. Chem. Phys..

[cit21] Aragones J. L., Sanz E., Valeriani C., Vega C. (2012). J. Chem. Phys..

[cit22] Dupont J. (2004). J. Braz. Chem. Soc..

[cit23] Paschoal V. H., Faria L. F. O., Ribeiro M. C. C. (2017). Chem. Rev..

[cit24] Endo T., Murata H., Imanari M., Mizushima N., Seki H., Sen S., Nishikawa K. (2013). J. Phys. Chem. B.

[cit25] Endo T., Higuchi T., Kimura Y. (2020). Bull. Chem. Soc. Jpn..

[cit26] Hunt P. A. (2007). J. Phys. Chem. B.

[cit27] Frisch M. J. , Trucks G. W., Schlegel H. B., Scuseria G. E., Robb M. A., Cheeseman J. R., Scalmani G., Barone V., Mennucci B., Petersson G. A., Nakatsuji H., Caricato M., Li X., Hratchian H. P., Izmaylov A. F., Bloino J., Zheng G., Sonnenberg J. L., Hada M., Ehara M., Toyota K., Fukuda R., Hasegawa J., Ishida M., Nakajima T., Honda Y., Kitao O., Nakai H., Vreven T., Montgomery Jr J. A., Peralta J. E., Ogliaro F., Bearpark M., Heyd J. J., Brothers E., Kudin K. N., Staroverov V. N., Keith T., Kobayashi R., Normand J., Raghavachari K., Rendell A., Burant J. C., Iyengar S. S., Tomasi J., Cossi M., Rega N., Millam J. M., Klene M., Knox J. E., Cross J. B., Bakken V., Adamo C., Jaramillo J., Gomperts R., Stratmann R. E., Yazyev O., Austin A. J., Cammi R., Pomelli C., Ochterski J. W., Martin R. L., Morokuma K., Zakrzewski V. G., Voth G. A., Salvador P., Dannenberg J. J., Dapprich S., Daniels A. D., Farkas O., Foresman J. B., Ortiz J. V., Cioslowski J. and Fox D. J., Gaussian 09, Gaussian, Inc., Wallingford CT, 2010

[cit28] Becke A. D. (1993). J. Chem. Phys..

[cit29] Lee C., Yang W., Parr R. G. (1988). Phys. Rev. B.

[cit30] Miehlich B., Savin A., Stoll H., Preuss H. (1989). Chem. Phys. Lett..

[cit31] Grimme S., Antony J., Ehrlich S., Krieg H. (2010). J. Chem. Phys..

[cit32] Marekha B. A., Kalugin O. N., Idrissi A. (2015). Phys. Chem. Chem. Phys..

[cit33] Van Der Spoel D., Lindahl E., Hess B., Groenhof G., Mark A. E., Berendsen H. J. C. (2005). J. Comput. Chem..

[cit34] Kutzner C., Páll S., Fechner M., Esztermann A., de Groot B. L., Grubmüller H. (2019). J. Comput. Chem..

[cit35] Darden T., York D., Pedersen L. (1993). J. Chem. Phys..

[cit36] Essmann U., Perera L., Berkowitz M. L., Darden T., Lee H., Pedersen L. G. (1995). J. Chem. Phys..

[cit37] Wang J., Wolf R. M., Caldwell J. W., Kollman P. A., Case D. A. (2004). J. Comput. Chem..

[cit38] Chen H., Yan T., Voth G. A. (2009). J. Phys. Chem. A.

[cit39] Reichert W. M., Holbrey J. D., Swatloski R. P., Gutowski K. E., Visser A. E., Nieuwenhuyzen M., Seddon K. R., Rogers R. D. (2007). Cryst. Growth Des..

[cit40] Choudhury A. R., Winterton N., Steiner A., Cooper A. I., Johnson K. A. (2005). J. Am. Chem. Soc..

[cit41] Endo T., Morita T., Nishikawa K. (2011). Chem. Phys. Lett..

[cit42] Domańska U., Pobudkowska A., Eckert F. (2006). Green Chem..

