# Supplementary material for: Origin of low melting point of ionic liquids: dominant role of entropy
Source: Chem Sci. 2022 Jun 8;13(25):7560–5. doi: 10.1039/d2sc02342c (PMC9241968; doi:10.1039/d2sc02342c)
Supplement: SC-013-D2SC02342C-s001 [file SC-013-D2SC02342C-s001.pdf]

Electronic Supplementary Material (ESI) for Chemical Science.
This journal is © The Royal Society of Chemistry 2022

Supplementary Information

Origin of Low Melting Point of Ionic Liquids: Dominant Role of Entropy

Takatsugu Endo, Kouki Sunada, Hiroki Sumida, and Yoshifumi Kimura

Corresponding Author: Takatsugu Endo
Email: taendo@mail.doshisha.ac.jp

This PDF file includes:

Supplementary text
Figures S1 to S15
Tables S1 to S12
SI References

Supplementary Information Text

Details of IL synthesis.
1,3-dimethylimidazolium iodide ([C_1mim]I): 1-Methylimidazole (0.268 mol, 22.0 g) and a slight excess amount of iodomethane (0.295 mol, 41.9 g) were dissolved in ethyl acetate. The solution was stirred under an inert atmosphere for 1 hour in an ice bath. The precipitate was washed with ethyl acetate five times, and subsequently recrystallized with acetone. A colorless crystal of [C_1mim]I was obtained via filtration (yield: 94%).
^{1}H-NMR (DMSO-d_6): δ (in ppm) = 9.07 (1H, s, NC***H***N), 7.69 (2H, t, NC***H***C***H***), 3.82 (6H, s, (NC$\boldsymbol{H}_3$)$_2$)
^{13}C-NMR (DMSO-d_6): δ (in ppm) = 137.5 (s, N***C***HN), 123.9 (s, N***C***H***C***H), 36.4 (s, N***C***H_3)
Water content: 20 ppm

1,3-dimethylimidazolium nitrate ([C_1mim]NO_3): $AgNO_3$ (0.031 mol, 5.27 g) was added into [C_1mim]I (0.034 mol, 7.62 g) aqueous solution. The solution was stirred for 2 hours with light shielding. After the reaction, the precipitant (AgI) was removed by filtration, and the solvent was removed by evaporation. The obtained solid was dissolved in dichloromethane, which produces white precipitant (residual AgI). The precipitant and dichloromethane were removed by filtration and evaporation, respectively. This residual byproduct-removing process was repeated until no precipitant was observed. After the final evaporation, the obtained solid was recrystallized with acetonitrile. A colorless crystal was then obtained via filtration (yield: 64%).
^{1}H-NMR (DMSO-d_6): δ (in ppm) = 9.08 (1H, s, NC***H***N), 7.67 (2H, t, NC***H***C***H***), 3.82 (6H, s, (NC$\boldsymbol{H}_3$)$_2$)
^{13}C-NMR (DMSO-d_6): δ (in ppm) = 137.7 (s, N***C***HN), 123.9 (s, N***C***H***C***H), 36.1 (s, N***C***H_3)
Water content: 60 ppm
I^- content: 320 ppm

1,3-dimethylimidazolium acetate ([C_1mim]CH_3CO_2): By passing acetic acid (0.235 mol, 14.1 g) aqueous solution through a column filled with the ion exchange resin (Amberlite IRN78, hydroxide form, 55 ml), the ion exchange resin of $CH_3CO_2^-$ form was obtained. Subsequently, [C_1mim]I (0.031 mol, 6.95 g) dissolved in distilled water was passed through the column to produce [C_1mim]CH_3CO_2 aqueous solution. No detectable iodide salt was confirmed in the solution by the $AgNO_3$ test. Water was removed from the solution by evaporation and subsequent vacuuming. After washing with ethyl acetate, the obtained solid was recrystallized with acetonitrile to give a colorless crystal (yield: 90%).
^{1}H-NMR (DMSO-d_6): δ (in ppm) = 10.18 (1H, s, NC***H***N), 7.83 (2H, t, NC***H***C***H***), 3.84 (6H, s, (NC$\boldsymbol{H}_3$)$_2$), 1.54 (3H, s, C$\boldsymbol{H}_3$COO)
^{13}C-NMR (DMSO-d_6): δ (in ppm) = 173.7 (s, CH_3***C***OO), 139.1 (s, N***C***HN), 123.9 (s, N***C***H***C***H), 35.8 (s, N***C***H_3), 26.9 (s, ***C***H_3COO)
Water content: 530 ppm.

1,3-dimethylimidazolium trifluoroacetate ([C_1mim]CF_3CO_2): 1-Methylimidazole (0.050 mol, 4.11 g) and a slight excess amount of methyl trifluoroacetate (0.055 mol, 7.04 g) were dissolved in ethyl acetate. The solution was stirred under an inert atmosphere for 1 day at 373 K. The obtained solid was washed with ethyl acetate five times, and subsequently recrystallized with acetonitrile. A colorless crystal of [C_1mim]CF_3CO_2 was obtained via filtration (yield: 61%).
^{1}H-NMR (DMSO-d_6): δ (in ppm) = 9.16 (1H, s, NC***H***N), 7.69 (2H, t, NC***H***C***H***), 3.82 (6H, s, (NC$\boldsymbol{H}_3$)$_2$)
^{13}C-NMR (DMSO-d_6): δ (in ppm) = 158.5 (q, CF_3***C***OO), 137.7 (s, N***C***HN), 124.0 (s, N***C***H***C***H), 115.9 (s, ***C***F_3COO), 36.1 (s, N***C***H_3)
^{19}F-NMR (DMSO-d_6): δ (in ppm) = −73.5 (3F, s, C$\boldsymbol{F}_3$COO)
Water content: 40 ppm.

1,3-dimethylimidazolium mesylate ([C_1mim]CH_3SO_3): 1-Methylimidazole (0.100 mol, 8.21 g) and a slight excess amount of methyl methanesulfonate (0.110 mol, 12.1 g) were dissolved in ethyl acetate. The solution was stirred under an inert atmosphere for 1 day in an ice bath. The obtained

solid was washed with ethyl acetate five times, and subsequently recrystallized with acetonitrile. A colorless crystal of $[C_1mim]CH_3SO_3$ was obtained via filtration (yield: 94%).
^{1}H-NMR (DMSO-*d*$_6$): *δ* (in ppm) = 9.13 (1H, s, NC***H***N), 7.69 (2H, t, NC***H***C***H***), 3.82 (6H, s, (NC***H***$_3$)$_2$), 2.32 (3H, s, CH_3SO_3)
^{13}C-NMR (DMSO-*d*$_6$): *δ* (in ppm) = 137.8 (s, N***C***HN), 124.0 (s, N***C***H***C***H), 40.4 (s, CH_3SO_3) 36.1 (s, N***C***H$_3$)
Water content: 120 ppm.

1,3-dimethylimidazolium trifluoromethanesulfonate ($[C_1mim]CF_3SO_3$): 1-Methylimidazole (0.050 mol, 4.11 g) and a slight excess amount of methyl trifluoromethanesulfonate (0.055 mol, 9.03 g) were dissolved in ethyl acetate. The solution was stirred under an inert atmosphere for 1 day in an ice bath. The solution was evaporated to remove ethyl acetate. The obtained solid was washed with diethyl ether five times, and subsequently recrystallized with acetonitrile. A colorless crystal was obtained as the final product (yield: 95%).
^{1}H-NMR (DMSO-*d*$_6$): *δ* (in ppm) = 8.97 (1H, s, NC***H***N), 7.63 (2H, t, NC***H***C***H***), 3.81 (6H, s, (NC***H***$_3$)$_2$)
^{13}C-NMR (DMSO-*d*$_6$): *δ* (in ppm) = 137.5 (s, N***C***HN), 123.9 (s, N***C***H***C***H), 119.1 (s, CF_3SO_3), 36.1 (s, N***C***H$_3$)
^{19}F-NMR (DMSO-*d*$_6$): *δ* (in ppm) = −77.8 (3F, s, CF_3SO_3)
Water content: 30 ppm.

1,3-dimethylimidazolium tosylate ($[C_1mim][OTs]$): 1-Methylimidazole (0.050 mol, 4.11 g) and a slight excess amount of methyl *p*-toluenesulfonate (0.055 mol, 10.2 g) were dissolved in ethyl acetate. The solution was stirred under an inert atmosphere for 1 day at room temperature. The obtained solid was washed with ethyl acetate five times, and subsequently recrystallized with acetonitrile. A colorless crystal of $[C_1mim][OTs]$ was obtained via filtration (yield: > 99 %).
^{1}H-NMR (DMSO-*d*$_6$): *δ* (in ppm) = 9.04 (1H, s, NC***H***N), 7.65 (2H, t, NC***H***C***H***), 7.48 (2H, d, CH$_3$CC***H***), 7.09 (2H, d, C***H***CSO$_3$), 3.78 (6H, s, (NC***H***$_3$)$_2$), 2.25 (3H, s, CC***H***$_3$)
^{13}C-NMR (DMSO-*d*$_6$): *δ* (in ppm) = 146.1 (s, C***C***H$_3$), 138.3 (s, CH***C***CH$_3$), 137.7 (s, N***C***HN), 128.7 (d, CH$_3$C***C***H), 126.0 (d, ***C***HCSO$_3$), 123.9 (s, N***C***H***C***H), 36.1 (s, N***C***H$_3$), 21.3 (q, ***C***SO$_3$)
Water content: 160 ppm.

1,3-dimethylimidazolium thiocyanate ($[C_1mim]SCN$): Anion exchange from $[C_1mim]I$ (0.045 mol, 10.1 g) to $[C_1mim]OH$ was conducted with ion exchange resin (Amberlite IRN78, hydroxide form, 75 ml). Complete anion exchange was confirmed by the $AgNO_3$ test. $[C_1mim]OH$ was neutralized with HCl to give $[C_1mim]Cl$ aqueous solution. Water in the solution was removed by evaporation and subsequent vacuuming. After recrystallization with acetonitrile, a colorless crystal of $[C_1mim]Cl$ was obtained via filtration (yield: 68%). Then, NaSCN (0.024 mol, 1.95 g) and $[C_1mim]Cl$ (0.022 mol, 2.92 g) were dissolved into distilled water, and stirred at room temperature for 1 day. After evaporation of the solution, dichloromethane was added into crude $[C_1mim]SCN$ to produce white precipitant (NaCl). The precipitant and dichloromethane were removed by filtration and evaporation, respectively. This process was repeated until no precipitant was obtained. After the final evaporation, $[C_1mim]SCN$ was obtained as a pale yellow liquid (yield: 64%).
^{1}H-NMR (DMSO-*d*$_6$): *δ* (in ppm) = 8.96 (1H, s, NC***H***N), 7.62 (2H, t, NC***H***C***H***), 3.83 (6H, s, (NC***H***$_3$)$_2$)
^{13}C-NMR (DMSO-*d*$_6$): *δ* (in ppm) = 137.5 (s, N***C***HN), 130.5 (s, S***C***N), 123.9 (s, N***C***H***C***H), 36.3 (s, N***C***H$_3$)
Water content: 140 ppm.
Na^+ content: 400 ppm

1,3-dimethylimidazolium dicyanamide ($[C_1mim]N(CN)_2$): The synthetic procedure was the same as that of $[C_1mim]SCN$ except that $NaN(CN)_2$ was used instead of NaSCN. The $[C_1mim]N(CN)_2$ was recrystallized with acetonitrile. After filtration and subsequent evaporation, $[C_1mim]N(CN)_2$ was obtained as a pale yellow liquid (supercooled liquid) at room temperature (yield: 73%).

^{1}H-NMR (DMSO-d_6): δ (in ppm) = 8.98 (1H, s, NC***H***N), 7.61 (2H, t, NC***H***C***H***), 3.81 (6H, s, (NC$\boldsymbol{H}_3$)$_2$)
^{13}C-NMR (DMSO-d_6): δ (in ppm) = 137.5 (s, N***C***HN), 123.9 (s, N***C***H***C***H), 119.6 (s, N***C***N), 36.2 (s, N***C***H_3)
Water content: 90 ppm.
Na^+ content: 320 ppm

1,3-dimethylimidazolium tricyanomethanide ([C_1mim]C$(CN)_3$): The synthetic procedure was the same as that of [C_1mim]SCN except that NaC$(CN)_3$ was used instead of NaSCN. The final product was a pale yellow liquid (supercooled liquid) at room temperature (yield: 68%).
^{1}H-NMR (DMSO-d_6): δ (in ppm) = 8.97 (1H, s, NC***H***N), 7.59 (2H, t, NC***H***C***H***), 3.80 (6H, s, (NC$\boldsymbol{H}_3$)$_2$)
^{13}C-NMR (DMSO-d_6): δ (in ppm) = 137.5 (s, N***C***HN), 123.9 (s, N***C***H***C***H), 121.0 (s, C***C***N), 36.2 (s, N***C***H_3), 5.3 (s, ***C***CN)
Water content: 40 ppm.
Na^+ content: 70 ppm.

Brief Theoretical background of two-phase thermodynamic (2PT) approach. The theory of 2PT is briefly described as follows (for details, please see the references[1, 2]). The total kinetic entropy of a molecule in a liquid state can be divided into translational (S_{tra}), rotational (S_{rot}), and intramolecular vibrational (S_{vib}) contributions.

$$S = S_{\mathrm{tra}} + S_{\mathrm{rot}} + S_{\mathrm{vib}} \quad \text{(S1)}$$

In 2PT, S_{tra} and S_{rot} where diffusive motions are included are considered as a sum of gaseous and solid components.

$$S_{\mathrm{tra}} = S_{\mathrm{tra}}^{\mathrm{g}} + S_{\mathrm{tra}}^{\mathrm{s}} \quad \text{(S2)}$$

$$S_{\mathrm{rot}} = S_{\mathrm{rot}}^{\mathrm{g}} + S_{\mathrm{rot}}^{\mathrm{s}} \quad \text{(S3)}$$

The estimation of these entropies is based on density of states function $g(v)$ which is the Fourier transform of the velocity autocorrelation function $C(t)$ of a molecule,

$$g(v) = \frac{2}{kT}\lim_{\tau\to\infty}\int_{-\tau}^{\tau} C(t)e^{-i2\pi vt}dt \quad \text{(S4)}$$

where k is the Boltzmann constant, T is temperature, and v is the frequency. $C(t)$ is the sum of the mass-weighted velocity autocorrelation function of atoms,

$$C(t) = \sum_{\mathrm{j=1}}^{N}\sum_{\mathrm{k=1}}^{3} m_{\mathrm{j}} c_{\mathrm{j}}^{\mathrm{k}}(t) \quad \text{(S5)}$$

where m is the mass of an atom j and N is the total number of atoms of the systems. Same as the entropy, $g(v)$ is divided into translational ($g_{\mathrm{tra}}(v)$), rotational ($g_{\mathrm{rot}}(v)$), and intramolecular vibrational ($g_{\mathrm{vib}}(v)$) components.

$$g(v) = g_{\mathrm{tra}}(v) + g_{\mathrm{rot}}(v) + g_{\mathrm{vib}}(v) \quad \text{(S6)}$$

The functions $g_{\mathrm{tra}}(v)$ and $g_{\mathrm{rot}}(v)$ are determined from autocorrelation functions of center-of-mass velocity and angular velocity of the molecule of interest, respectively. $g_{\mathrm{vib}}(v)$ is obtained by the deduction of $g_{\mathrm{tra}}(v)$ and $g_{\mathrm{rot}}(v)$ from the total density of states function. $g_{\mathrm{tra}}(v)$ contains gaseous and solid components.

$$g_{\mathrm{tra}}(v) = g_{\mathrm{tra}}^{g}(v) + g_{\mathrm{tra}}^{s}(v) \quad \text{(S7)}$$

$g^{\mathrm{g}}_{\mathrm{tra}}(v)$ is expressed by employing a hard-sphere model as,

$$g_{\text{tra}}^{\text{g}}(v)=\frac{g_{\text{tra}}(0)}{1+\left(\frac{\pi g_{\text{tra}}(0)v}{6f_{\text{tra}}N}\right)^2} \tag{S8}$$

where f_{tra} is the translational “fluidicity”. Because “fluidicity” expresses the fraction of the hard-sphere (gaseous) component in the overall system, the integral of $g^{\text{g}}_{\text{tra}}(v)$ corresponds to $3Nf_{\text{tra}}$. f_{tra} can be numerically derived with the following equations,

$$2\Delta_{\text{tra}}^{-9/2}f_{\text{tra}}^{15/2}-6\Delta_{\text{tra}}^{-3}f_{\text{tra}}^{5}-\Delta_{\text{tra}}^{-3/2}f_{\text{tra}}^{7/2}+6\Delta_{\text{tra}}^{-3/2}f_{\text{tra}}^{5/2}+2f_{\text{tra}}-2=0 \tag{S9}$$

$$\Delta_{\text{tra}}=\frac{2g_{\text{tra}}(0)}{9N}\left(\frac{\pi kT}{m}\right)^{1/2}\left(\frac{N}{V}\right)^{1/3}\left(\frac{6}{\pi}\right)^{2/3} \tag{S10}$$

where Δ is the dimensionless diffusivity constant and V is the system volume. The density of states at zero frequency $g_{\text{tra}}(0)$ can be determined directly from $g_{\text{tra}}(v)$ or via diffusion coefficient D of molecule.

$$g_{\text{tra}}(0)=\frac{12mND}{kT} \tag{S11}$$

Based on the Carnahan-Starling equation of state, the analytical form of the gaseous translational entropy is expressed as,

$$S_{\text{tra}}^{\text{g}}=\frac{5}{2}k+k\ln\left[\left(\frac{2\pi mkT}{h^2}\right)^{3/2}\frac{V}{f_{\text{tra}}N}Z\right]+\frac{y(3y-4)}{(1-y)^2}k \tag{S12}$$

$$Z=\frac{1+y+y^2-y^3}{(1-y)^3} \tag{S13}$$

$$y=\frac{f_{\text{tra}}^{5/2}}{\Delta^{3/2}} \tag{S14}$$

where h is the Planck constant, y is the hard-sphere packing fraction, and Z is the compressibility. The estimation of solid translational entropy is based on the harmonic oscillator model.

$$S_{\text{tra}}^{\text{s}}=k\ln Q_{\text{tra}}+\frac{1}{\beta}\left(\frac{\partial\ln Q_{\text{tra}}}{\partial T}\right)_{N,V} \tag{S15}$$

$$\beta=\frac{1}{kT} \tag{S16}$$

In the harmonic oscillator model, the canonical partition function of translation Q_{tra} was expressed as,

$$\ln Q_{\text{tra}}=\int_0^\infty g_{\text{tra}}^{\text{s}}(v)\ln q_{\text{HO}}(v)dv \tag{S17}$$

$$q_{\text{HO}}(v)=\frac{e^{-\beta hv/2}}{1-e^{-\beta hv}} \tag{S18}$$

S_{rot} was estimated in a similar manner as S_{tra}. Since S_{vib} contains no diffusive motion, it is determined only in the harmonic oscillator framework.

Melting point (T_{m}), fusion enthalpy ($\Delta_{\text{fus}}H$), and fusion entropy ($\Delta_{\text{fus}}S$) estimations. To estimate T_{m}, $\Delta_{\text{fus}}H$, and $\Delta_{\text{fus}}S$, first, Helmholtz energy difference between liquid and crystal ($\Delta_{\text{ref}}A$) at a certain reference temperature T_{ref} is required. T_{ref} of NaCl, $[C_2mim]PF_6$, and $[C_4mim]PF_6$ were

set to be 1100 K, 380 K, and 340 K, respectively. Calculations of $\Delta_{ref}A$ were conducted based on a thermodynamic integration, called the pseudosupercritical path (PSCP) cycle[3, 4] where $\Delta_{ref}A$ was derived as the sum of four ΔA values (Figure S3).

$$\Delta_{\mathrm{ref}} A = \Delta_1 A + \Delta_2 A + \Delta_3 A + \Delta_4 A \tag{S19}$$

Except for $\Delta_3 A$, the Helmholtz energy difference in the PSCP cycle is expressed as,

$$\Delta A = \int_0^1 \left\langle \frac{dU}{d\lambda} \right\rangle_\lambda d\lambda \tag{S20}$$

where λ is the alchemical variable ranging from 0 to 1 and U is the potential energy. Starting from the "crystal" state, it is first transformed to the "weak crystal" state. In this step, both LJ (U_{LJ}) and Coulombic (U_{Coul}) potentials are weakened, and a tether potential (U_{tether}) emerges.

$$U_1 = (1-0.9\lambda)U_{\mathrm{LJ}} + (1-0.9\lambda)^2 U_{\mathrm{Coul}} + \lambda U_{\mathrm{tether}} + U_{\mathrm{bonded}} \tag{S21}$$

$$U_{\mathrm{tether}} = \sum_{\mathrm{i}} \sum_{\mathrm{j}} a_{\mathrm{ij}} e^{-b_{\mathrm{ij}} r_{\mathrm{ij}}^2} \tag{S22}$$

The tether potential that binds atoms to lattice points has the Gaussian function form. It was applied for both Na^+ and Cl^- of NaCl. For ILs, the C and N atoms of the cation and the P atom of the anion were used for U_{tether}. The constant a of the cation atoms was 16.0254 kJ mol^{-1}, and that of the anion atom was 14.0789 kJ mol^{-1}.[4] The value of 90 nm^{-2} was used for the constant b of every atom.[4] U_{bonded} is the potential for intramolecular bonds, angles, dihedral angles, and improper angles, which are constant during the PSCP cycle.

In step 2, the "weak crystal" is transformed into "weak dense fluid" by removing the tether potential

$$U_2 = 0.1U_{\mathrm{LJ}} + 0.01U_{\mathrm{Coul}} + (1-\lambda)U_{\mathrm{tether}} + U_{\mathrm{bonded}} \tag{S23}$$

The "weak dense fluid" is then transformed into the "weak liquid". In this step, the cell volume is changed from that of crystal (V_{cry}) to liquid (V_{liq}) while the potential is not varied. Therefore, the Helmholtz energy difference ($\Delta_3 A$) is,

$$\Delta_3 A = \int_{V_{\mathrm{cry}}}^{V_{\mathrm{liq}}} p dV \tag{S24}$$

In step 4, intermolecular potentials are retrieved to the original one, corresponding to the transformation from the "weak liquid" to the normal "liquid" states, as

$$U_4 = (0.1+0.9\lambda)U_{\mathrm{LJ}} + (0.1+0.9\lambda)^2 U_{\mathrm{Coul}} + U_{\mathrm{bonded}} \tag{S25}$$

The results from the PSCP cycle for NaCl, $[C_2mim]PF_6$, and $[C_4mim]PF_6$ are displayed in Figures S4–S6 and Table S6. The Gibbs energy difference at the reference temperature in the NPT ensemble was obtained from $\Delta_{ref}A$,

$$\Delta_{\mathrm{ref}} G = \Delta_{\mathrm{ref}} A + p\Delta V \tag{S26}$$

With the $\Delta_{ref}G$ value, it is now possible to estimate T_m where $\Delta G = 0$ via the Gibbs-Helmholtz equation

$$\int_{\Delta_{\mathrm{ref}} G}^{\Delta G} d\frac{\Delta G}{T} = \int_{T_{\mathrm{ref}}}^{T} -\frac{\Delta H}{T^2} dT \tag{S27}$$

The enthalpy differences between the crystal and liquid states obtained from the NPT MD simulations at various temperatures were fitted with a second-degree polynomial function. Then, equation (S27) becomes,

$$\frac{\Delta G}{T} - \frac{\Delta_{\mathrm{ref}} G}{T_{\mathrm{ref}}} = a\left(\frac{1}{T} - \frac{1}{T_{\mathrm{ref}}}\right) - b\ln\frac{T}{T_{\mathrm{ref}}} - c(T - T_{\mathrm{ref}}) \tag{S28}$$

where a, b, and c are the fitting constant. With T_m value where $\Delta G = 0$ (Figure S7) and $\Delta_{fus}H$ at the same temperature, $\Delta_{fus}S$ is obtained based on equation (1) in the main text. Obtained T_m,

$\Delta_{fus}H$, and $\Delta_{fus}S$ are summarized in Table S7 with reported experimental and MD values. A production run of 2 ns was applied for these simulations[4-7] with 0.1 ps data accumulations.

Conformational entropy (S_{confor}) estimation. $[C_2mim]^+$ is known to possess non-planar (n) and planar (p) conformations along the C-N-C-C dihedral angle (Figure S8A).[8, 9] By including the mirror-inverted conformation of the non-planar (n'), the cation has three conformers, which were also observed in our simulations (Figure S8B). In addition to the C-N-C-C dihedral angle, the N-C-C-C and C-C-C-C dihedral angles are present in $[C_4mim]^+$, which produce additional three conformers each, i.e., trans (t), gauche (g), and gauche' (g') (Figure S9A).[8, 10] In total, this cation has 3 × 3 × 3 = 27 conformations. Hereafter, for example, the conformation for $[C_4mim]^+$ with non-planar (C-N-C-C), gauche (N-C-C-C), and trans (C-C-C-C) is abbreviated as ngt. Conformational analyses were performed on the cations in $[C_2mim]PF_6$ or $[C_4mim]PF_6$ via 20 ns simulations with 2 ps data accumulations in the NVT ensemble. Once a population of each conformer is estimated from MD trajectories, conformational entropy (S_{confor}) was calculated,

$$S_{\mathrm{confor}} = -R\sum_{\mathrm{i}} p_{\mathrm{i}} \ln p_{\mathrm{i}} \qquad \text{(S29)}$$

where R is the gas constant and p_i is the population of the conformer i. The results are summarized in Tables S8 and S9.

Kinetic entropy (S_{kin}) estimation. The production runs for 2PT were executed with the velocity Verlet algorithm for 1 ns (a simulation time of 250 ps for 1 block) with 2 fs data acquisition in the NVT ensemble. The convergence of the 2PT method is known to fast (typically ca. 20 ps),[2, 11, 12] and we confirmed that 250 ps is long enough for $[C_4mim]PF_6$ (Figure S10). Translational, rotational, and vibrational density of states functions were obtained using the DoSPT program.[13] The results are displayed in Figures S11–S14, and the obtained numerical values are in Tables S10 and S11

Diffusion coefficient (D) estimation. For diffusion coefficient (D) estimations, production runs of 200 ns with 0.1 ps data acquisition were performed in the NVT ensemble. The diffusion coefficients of the ions were calculated from mean square displacement (MSD) with the Einstein's equation (Figure S15). The D values are listed in Table S12.

$$D = \left[\frac{1}{6t}\mathrm{MSD}\right]_{t\to\infty} \qquad \text{(S30)}$$

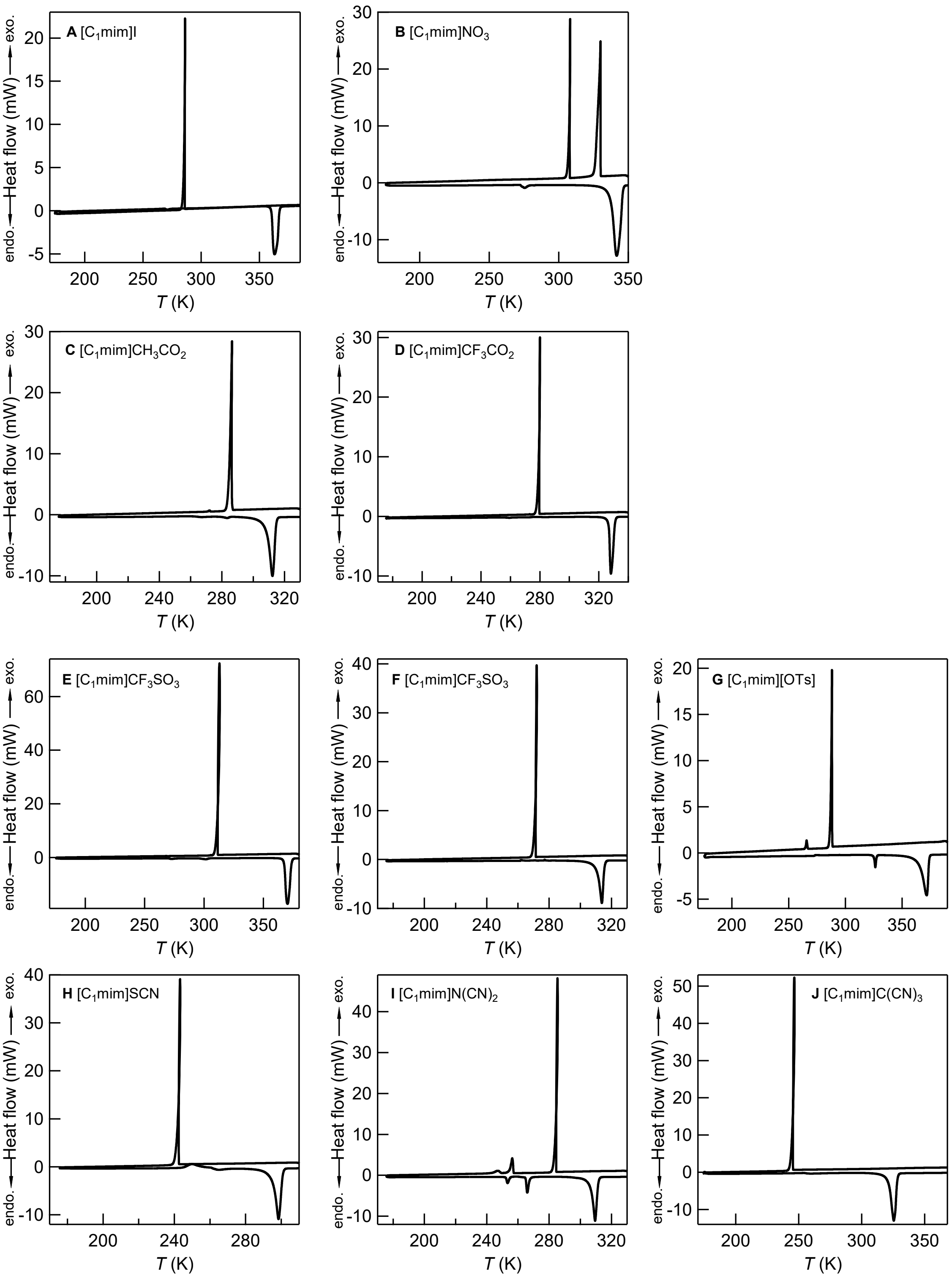

Fig. S1. DSC traces of $[C_1mim]X$.

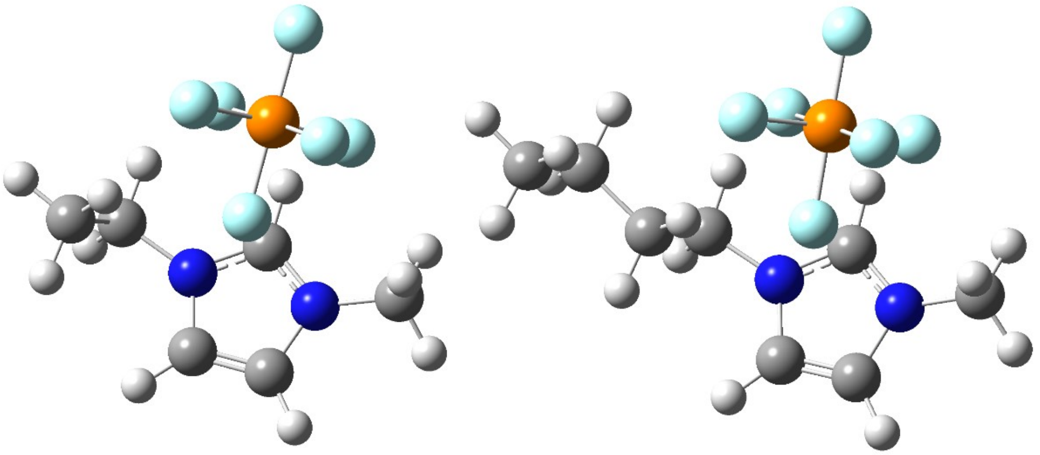

Fig. S2. Optimized local minima for ion pairs of $[C_2mim]PF_6$ (left) and $[C_4mim]PF_6$ (right) in the gas phase.

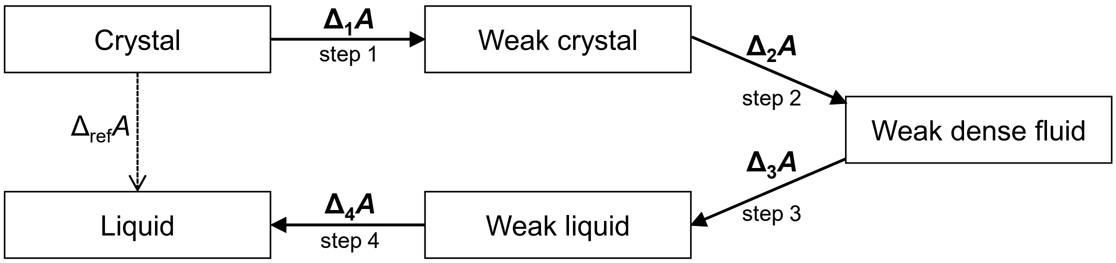

Fig. S3. Schematic of the PSCP cycle.

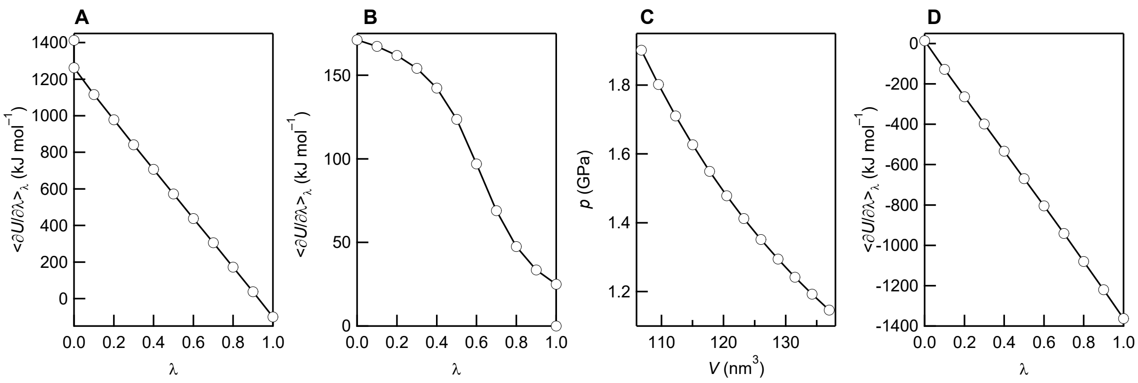

Fig. S4. Derivatives of the potential energy per ion pair of NaCl in (A) step 1, (B) step 2, and (D) step 4 along the PSCP cycle. (C) Pressure as a function of volume for step 3.

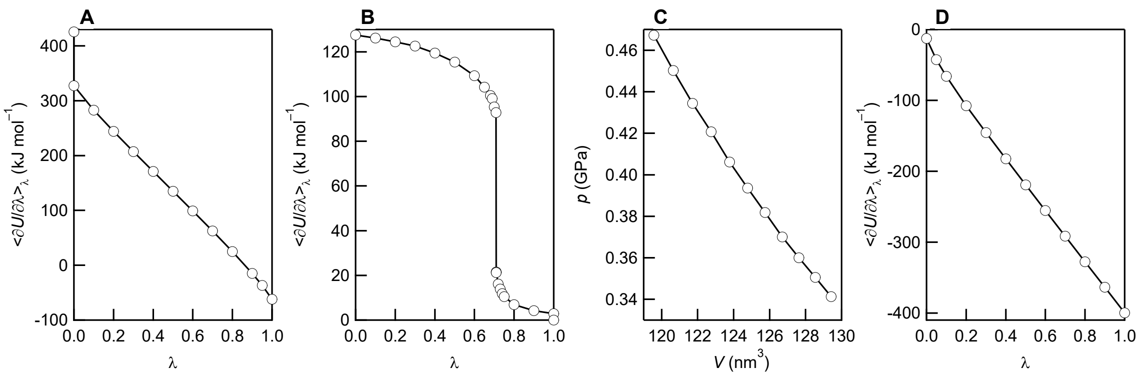

Fig. S5. Derivatives of the potential energy per ion pair of $[C_2mim]PF_6$ in (A) step 1, (B) step 2, and (D) step 3 along the PSCP cycle. (C) Pressure as a function of volume for step 3.

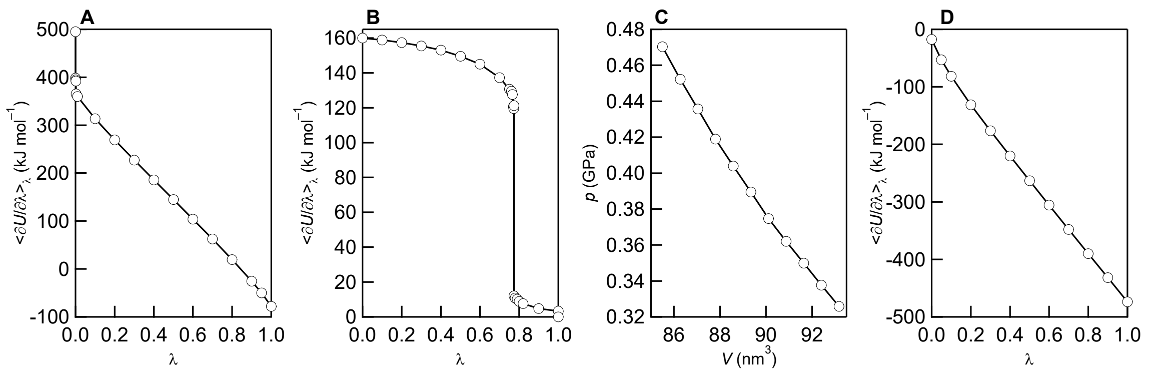

Fig. S6. Derivatives of the potential energy per ion pair of [C_4mim]PF_6 in (A) step 1, (B) step 2, and (D) step 3 along the PSCP cycle. (C) Pressure as a function of volume for step 3.

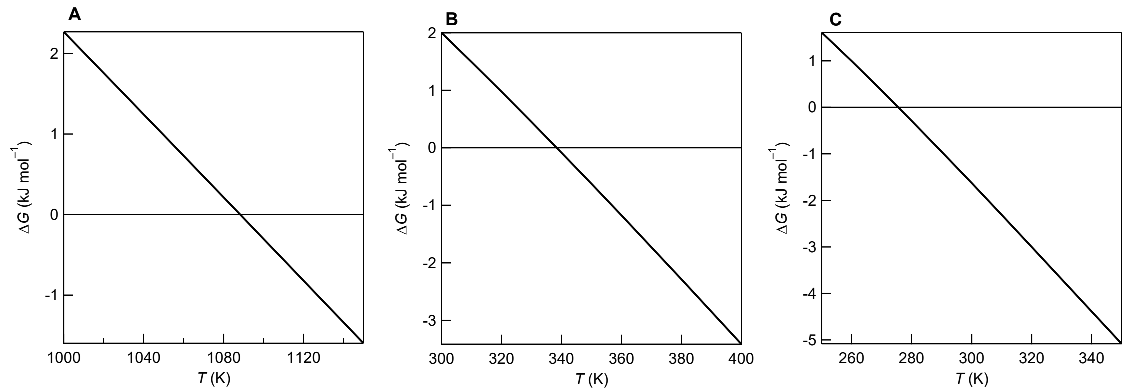

Fig. S7. Calculated Δ*G* versus temperature. (A) NaCl, (B) $[C_2mim]PF_6$, and (C) $[C_4mim]PF_6$.

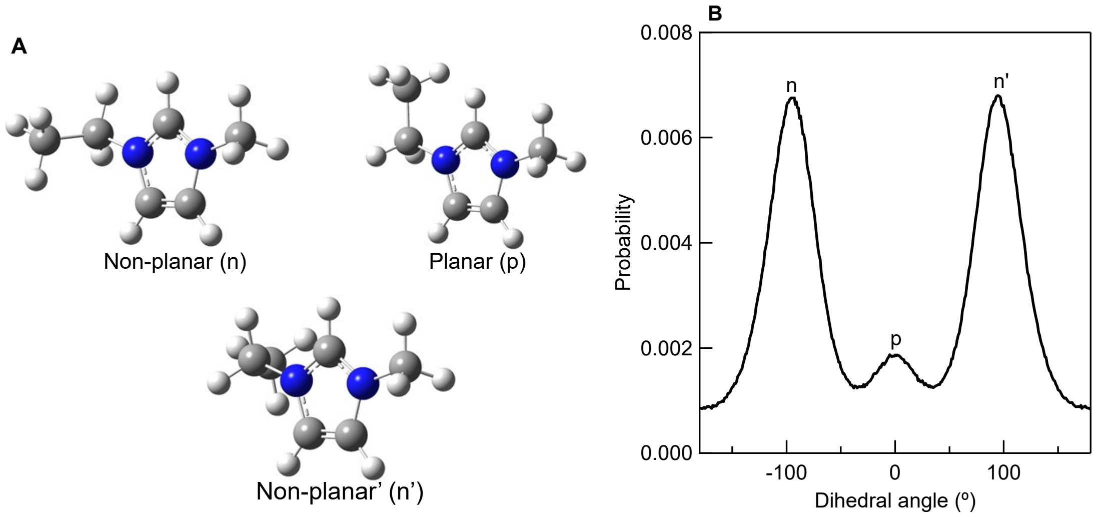

Fig. S8. (A) Structure of three conformers for $[C_2mim]^+$. (B) Dihedral angle distribution of C-N-C-C of $[C_2mim]^+$ in $[C_2mim]PF_6$ in the liquid state at 338 K.

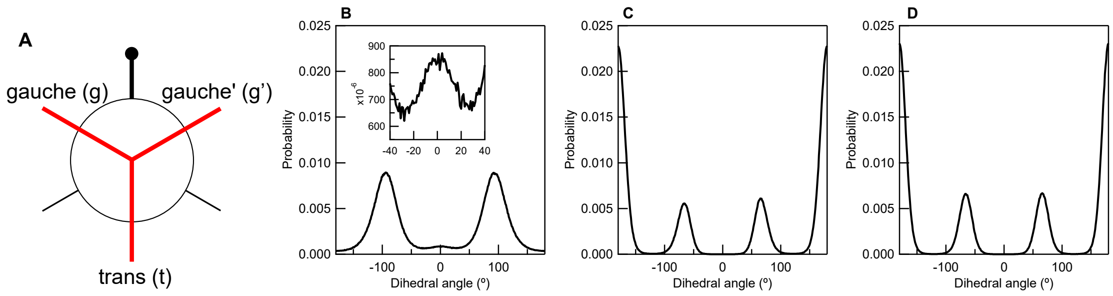

Fig. S9. (A) Newman projection for the trans, gauche, and gauche' conformers. Dihedral angle distributions of (B) C-N-C-C, (C) N-C-C-C, and (D) C-C-C-C of $[C_4mim]^+$ in $[C_4mim]PF_6$ in the liquid state at 275 K.

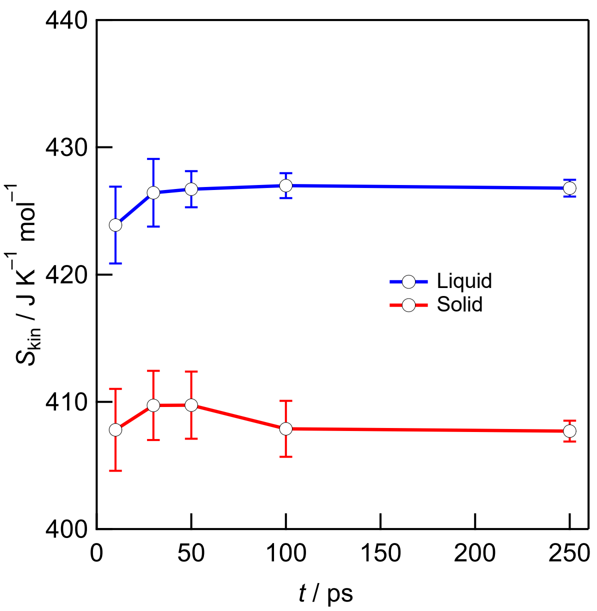

Fig. S10. Kinetic entropies estimated from the 2PT method for [C_4mim]PF_6 at 275 K as a function of simulation time.

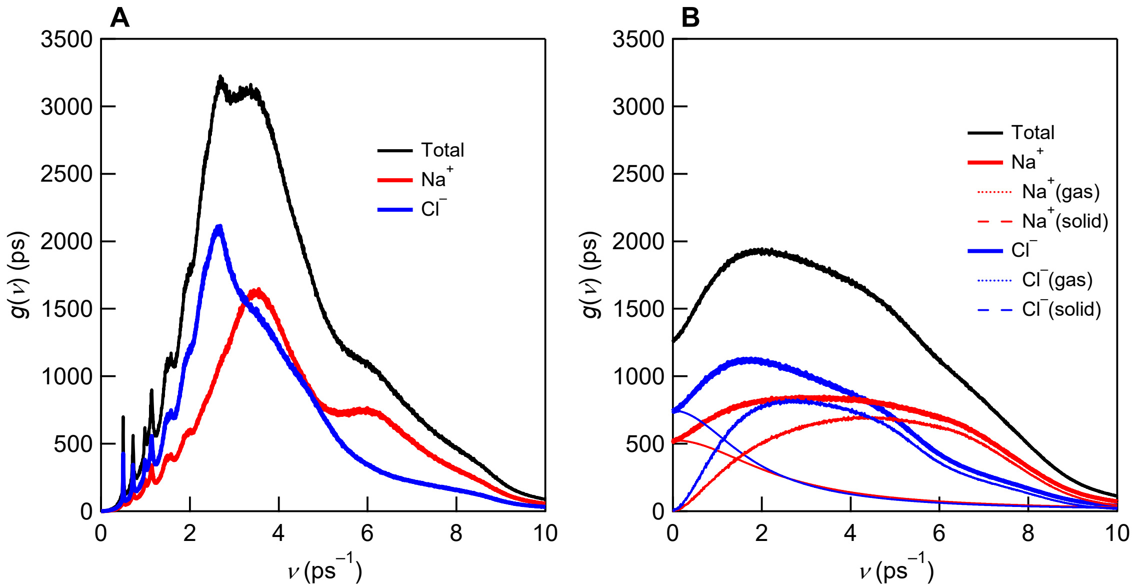

Fig. S11. Density of states functions of NaCl at 1089 K in the (A) crystal and (B) liquid states.

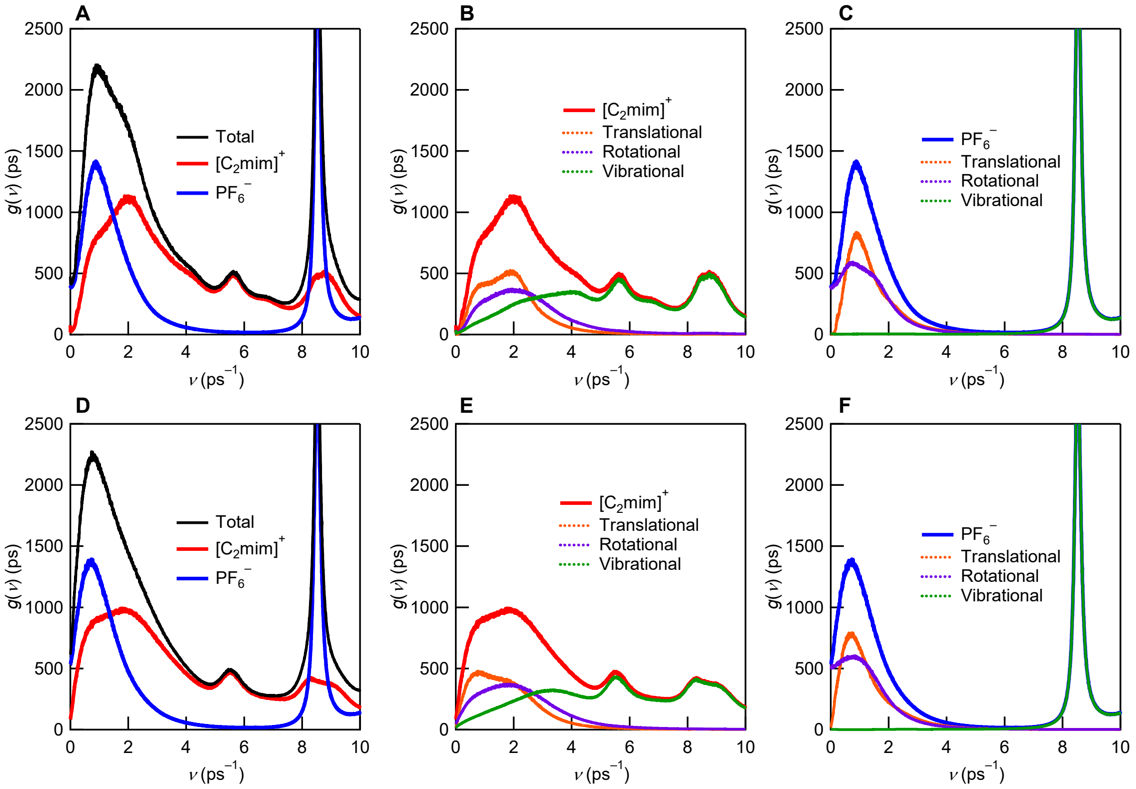

Fig. S12. Density of states functions of [C_2mim]PF_6 at 338 K in the (A–C) crystal and (D–F) liquid states.

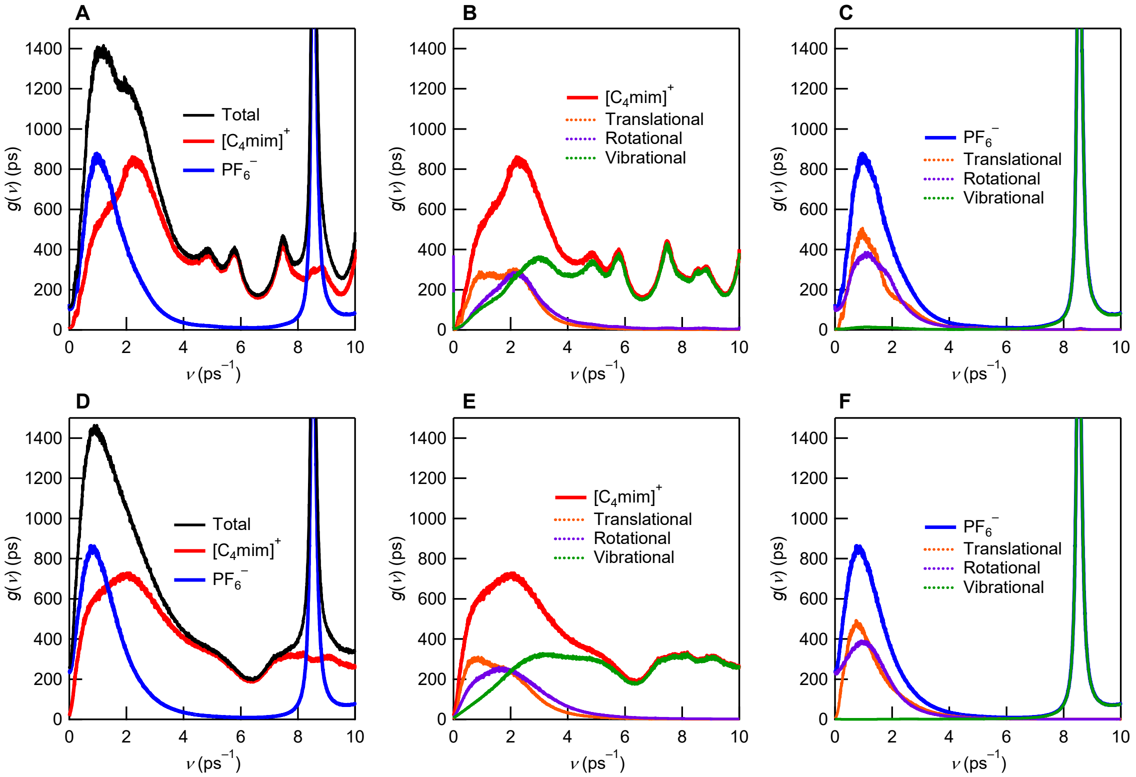

Fig. S13. Density of states functions of $[C_4mim]PF_6$ at 275 K in the (A–C) crystal and (D–F) liquid states.

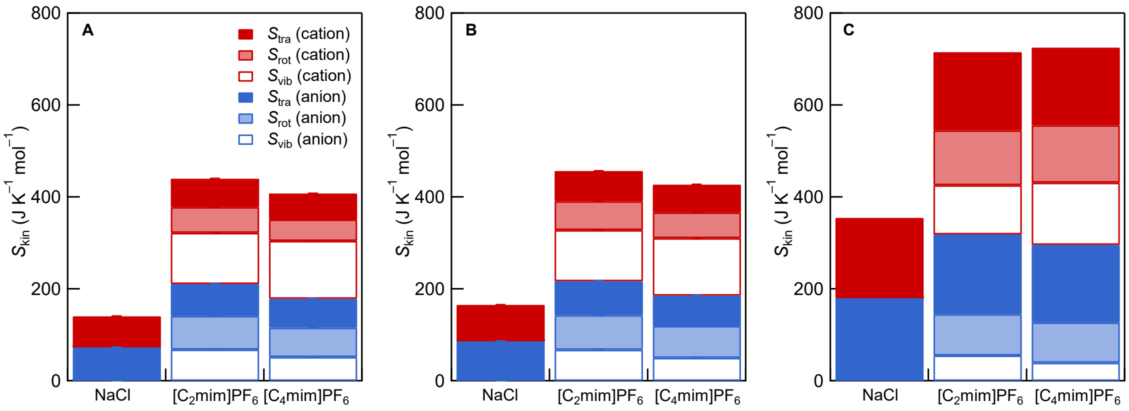

Fig. S14. Absolute kinetic entropies of NaCl (1089 K), [C_2mim]PF_6 (338 K), and [C_4mim]PF_6 (275 K). The results in the (A) crystal and (B) liquid states were obtained from the MD simulations (Tables S10 and S11) while that in the (C) gas state was estimated by the DFT calculations (Table S5) as the sum of the entropies of the isolated ions. The same symbols are used through (A–C).

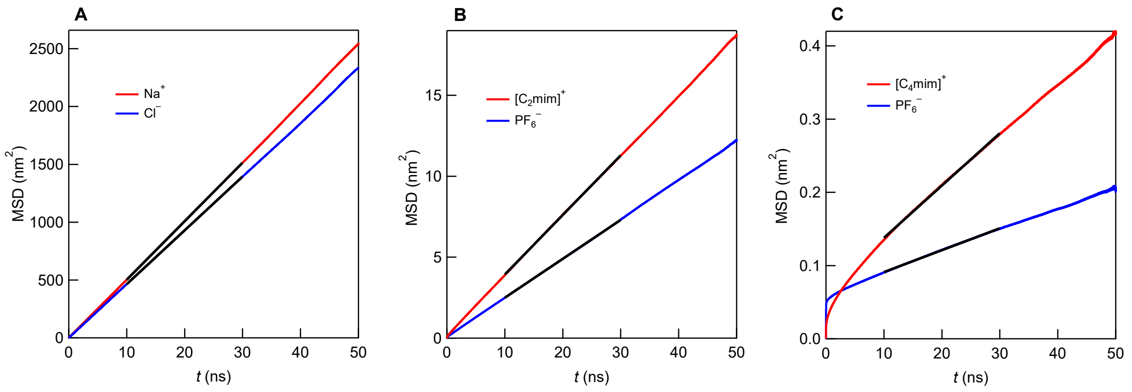

Fig. S15. Mean square displacements of the ions in (A) NaCl (1089 K), (B) $[C_2mim]PF_6$ (338 K), and (C) $[C_4mim]PF_6$ (275 K). Black lines are the linear fit from 10 ns to 30 ns.

Table S1. T_m, $\Delta_{fus}H$, and $\Delta_{fus}S$ data for ILs from the ILThermo database.[14, 15]

IL	T_m / K	$\Delta_{fus}H^a$ / kJ mol^{-1}	$\Delta_{fus}S^a$ / J K^{-1} mol^{-1}
Imidazolium			
1-Methylimidazolium nitrate[16]	343.6	19.24	56.00
1,3-Dimethylimidazolium methylsulfate[17]	308.9	16.58	53.67
1,3-Dimethylimidazolium bis(trifluoromethylsulfonyl)imide[18]	299	24.5	81.7
1-Ethyl-3-methylimidazolium chloride[19]	370.1	15.1	40.8
1-Ethyl-3-methylimidazolium thiocyanate[20]	267	27.0	101
1-Ethyl-3-methylimidazolium acetate[21]	370.85	30.2	81.4
1-Ethyl-3-methylimidazolium nitrate[22]	316.4	17.6	55.6
1-Ethyl-3-methylimidazolium bromide[23]	349.91	18.26	52.18
1-Ethyl-3-methylimidazolium tetrafluoroborate[24]	287.6	9.5	33.0
1-Ethyl-3-methylimidazolium tricyanomethanide[22]	274.9	12.6	45.8
1-Ethyl-3-methylimidazolium iodide[25]	351	15.488	44.125
1-Ethyl-3-methylimidazolium hexafluorophosphate[26]	334.2	17.7	53.2
1-Ethyl-3-methylimidazolium tetrachloroaluminate[19]	279.6	13.8	49.4
1-Ethyl-3-methylimidazolium 4-methylbenzenesulfonate[22]	328.2	20.1	61.2
1-Ethyl-3-methylimidazolium bis(trifluoromethylsulfonyl)imide[27]	271.44	21.89	80.64
1-Ethyl-3-methylimidazolium dimethylphosphate[22]	312.9	21.5	68.7
1-Ethyl-3-methylimidazolium 1,1,2,2-tetrafluoroethanesulfonate[28]	317.6	20.2	63.6
1-Ethyl-3-methylimidazolium trifluoromethanesulfonate[22]	262.6	11.7	44.6
1-Ethyl-3-methylimidazolium bis(fluorosulfonyl)imide[29]	260	9.3	35.8
1-Methyl-3-propylimidazolium bromide[30]	312.93	19.11	61.75
1-Methyl-3-propylimidazolium hexafluorophosphate[26]	311.8	14.1	45.2
1-Butyl-3-methylimidazolium chloride[31]	347.1	18	52
1-Butyl-3-methylimidazolium nitrate[32]	309.18	17.991	58.19
1-Butyl-3-methylimidazolium dicyanamide[33]	270.83	17.8	65.7
1-Butyl-3-methylimidazolium bromide[23]	351.35	22.88	65.12
1-Butyl-3-methylimidazolium trifluoroacetate[34]	296.41	19.14	64.59
1-Butyl-3-methylimidazolium iodide[30]	291.92	18.99	65.05
1-Butyl-3-methylimidazolium hexafluorophosphate[35]	283.5	19.601	69.139
1-Butyl-3-methylimidazolium trifluoromethanesulfonate[36]	291.46	20.18	69.24
1-Butyl-3-methylimidazolium tosylate[37]	343.89	21.573	62.732
1-Butyl-3-methylimidazolium octylsulfate[38]	307.6	12.7	41.3
1-Butyl-3-methylimidazolium bis(trifluoromethylsulfonyl)imide[39]	270.35	23.8	88
1-Butyl-3-methylimidazolium 2-methoxy-2-oxoacetate[40]	330.2	9	27
1-Hexyl-3-methylimidazolium bis(trifluoromethylsulfonyl)imide[41]	272.11	27.825	102.256
1-Methyl-3-octylimidazolium tetrafluoroborate[42]	245.81	15.31	62.27
1-Methyl-3-octyllimidazolium hexafluorophosphate[43]	272.3	12.9	47.4
1-Methyl-3-octylimidazolium bis(trifluoromethylsulfonyl)imide[27]	263.96	25.18	95.39
1-Methyl-3-octylimidazolium trifluoromethanesulfonate[36]	285.98	16.54	57.84
1-Nonyl-3-methylimidazolium hexafluorophosphate[26]	293	16.5	56.4
1-Decyl-3-methylimidazolium chloride[44]	311.2	30.9	99.3
1-Decyl-3-methylimidazolium bromide[45]	347.58	20.256	58.277
1-Decyl-3-methylimidazolium hexafluorophosphate[26]	307.1	19.4	63.3
1-Decyl-3-methylimidazolium bis(trifluoromethylsulfonyl)imide[46]	277.33	28.67	103.38
1-Decyl-3-methylimidazolium trifluoromethansulfate[47]	296.2	29.82	100.68
1-Dodecyl-3-methylimidazolium hexafluorophosphate[26]	326.5	24.5	75.2
1-Dodecyl-3-methylimidazolium bis(trifluoromethylsulfonyl)imide[48]	292.4	36	123
1-Tetradecyl-3-methylimidazolium bis(trifluoromethylsulfonyl)imide[46]	308.77	45.18	146.32
1-Hexadecyl-3-methylimidazolium bromide[45]	337.06	59.1	175.3
1-Hexadecyl-3-methylimidazolium bis(trifluoromethylsulfonyl)imide[46]	319.25	51.28	160.63

1-Methyl-3-octadecylimidazolium bis(trifluoromethylsulfonyl)imide[49]	328	53	162
1-Methyl-3-octadecylimidazolium tris(pentafluoroethyl)trifluorophosphate[49]	319	54	169
1-Methyl-3-octadecylimidazolium bis(nonafluorobutanesulfonyl)imide[49]	335	33	98
1-Docosyl-3-methylimidazolium bis(trifluoromethylsulfonyl)imide[49]	341	67	197
1-Isopropyl-3-methylimidazolium bis(fluorosulfonyl)imide[50]	269.2	14.3	53.3
1-Isopropyl-3-methylimidazolium bis(trifluoromethylsulfonyl)imide[50]	283.5	24.2	85.4
1-tert-Butyl-3-methylimidazolium bis(fluorosulfonyl)imide[50]	326.6	17.4	53.2
1-tert-Butyl-3-methylimidazolium bis(trifluoromethylsulfonyl)imide[50]	280.4	22.9	81.7
1-tert-Butyl-3-methylimidazolium bis(pentafluoroethylsulfonyl)imide[50]	294.3	16.7	56.6
1,3-Diethylimidazolium bis(trifluoromethylsulfonyl)imide[51]	262.6	20.4	77.7
1,2-Dimethyl-3-propylimidazolium bis(trifluoromethylsulfonyl)imide[24]	284.44	19.7	69.3
1-Butyl-2,3-dimethylimidazolium trifluoromethanesulfonate[52]	318	15	47
1-Butyl-2,3-dimethylimidazolium chloride[45]	326.57	14.413	44.134
1-Butyl-2,3-dimethylimidazolium bromide[45]	349.66	15.616	44.661
1-Decyl-2,3-dimethylimidazolium bromide[45]	341.35	23.923	70.083
1-Hexadecyl-2,3-dimethylimidazolium bromide[45]	371.7	50.8	136.7
1-Benzyl-3-methylimidazolium tetrafluoroborate[53]	346.2	19.1	55.2
1-Benzyl-3-methylimidazolium 1,1,2,2-tetrafluoroethanesulfonate[53]	315.4	23.6	74.8
1-Methyl-3-(2-phenylethyl)imidazolium bis(trifluoromethylsulfonyl)imide[54]	310.1	5.4	17.4
1-Methyl-3-(3-phenylpropyl)imidazolium hexafluorophosphate[54]	325.1	9.5	29.2
1-Methyl-3-(3-phenylpropyl)imidazolium bis(trifluoromethylsulfonyl)imide[54]	321.1	14	44
1-(2-Naphthylmethyl)-3-methylimidazolium bis(trifluoromethylsulfonyl)imide[55]	318.6	34.1	107.0
1,3-Dibenzylimidazolium bis(trifluoromethylsulfonyl)imide[55]	314.9	23.6	74.9
1,3-Bis(butoxymethyl)imidazolium tetrafluoroborate[56]	281.4	8.54	30.35
1,3-Bis((octyloxy)methyl)imidazolium bis(trifluoromethylsulfonyl)imide[56]	287.7	34.2	118.9
1-Isobutyl-3-methylimidazolium bis(trifluoromethylsulfonyl)imide[57]	256.9	6.8	26.5
1,3-Dihexyloxymethylimidazolium bis(trifluoromethylsulfonyl)imide[58]	273.8	16	58
1,3-Bis(hexyloxymethyl)imidazolium tetrafluoroborate[58]	309.5	12.8	41.4
1,3-Didecyl-2-methylimidazolium dicyanamide[59]	350.8	60.13	171.41
1-(3-Cyanopropyl)-3-methylimidazolium chloride[60]	363.5	19.1	52.6
1-Butyronitrile-3-methylimidazolium hexafluorophosphate[60]	345.2	17.5	50.7
1,3-Bis(decyloxy)-2-methylimidazolium bis(trifluoromethylsulfonyl)imide[56]	303.1	79.36	261.83
1-(2-Methoxyethyl)-3-ethylimidazolium perrhenate[61]	211.82	14.382	67.93
1-(Methoxymethyl)-3-methylimidazolium bis(trifluoromethylsulfonyl)imide[62]	273.0	22	81
1-(2-Ethoxyethyl)-3-methylimidazolium bis(trifluoromethylsulfonyl)imide[62]	265.6	14.1	53.1
Pyridinium			
1-Ethylpyridinium bis(trifluoromethylsulfonyl)imide[63]	303.6	18.9	62.3
1-Ethylpyridinium trifluoromethanesulfonate[64]	300.4	11.5	38.3
1-Propylpyridinium hexafluorophosphate[65]	370.99	6.83	18.41
1-Propylpyridinium bromide[66]	342.83	10.97	32.00
1-Butylpyridinium bis(trifluoromethylsulfonyl)imide[63]	299.1	27.9	93.3
1-Butylpyridinium trifluoromethanesulfonate[67]	301.4	12	40
1-Butylpyridinium tetrafluoroborate[67]	272.5	10.5	38.5
1-Pentylpyridinium hexafluorophosphate[65]	328.14	5.9	17.98
1-Pentylpyridinium bis(trifluoromethylsulfonyl)imide[63]	272.8	22.8	83.6
1-Methyl-3-propylpyridinium hexafluorophosphate[68]	311.2	15	48
1-Butyl-3-methylpyridinium 4-methylbenzenesulfonate[69]	323.7	11.34	35.03

1-Butyl-4-methylpyridinium tosylate[70]	324.86	14.33	44.11
1-Butyl-4-methylpyridinium bis(trifluoromethylsulfonyl)imide[71]	291.4	21.94	75.29
1-Hexyl-3-methylpyridinium chloride[72]	355.1	19.7	55.5
1-Hexyl-3-methylpyridinium trifluoromethanesulfonate[73]	337.76	41.968	124.254
1-Hexyl-3-methylpyridinium 4-methylbenzenesulfonate[71]	329.3	10.094	30.653
1-Octyl-3-methylpyridinium chloride[72]	352.3	14.9	42.3
1-Decyl-3-methylpyridinium chloride[72]	352.5	14.4	40.9
1-Dodecyl-3-methylpyridinium chloride[72]	360.8	37.1	102.8
1-Dodecyl-4-methylpyridinium chloride[74]	323.9	44.4	137.1
1-Tetradecyl-3-methylpyridinium chloride[72]	366.8	42.7	116.4
1-Butyl-3,5-dimethylpyridinium thiocyanate[75]	286.1	16.04	56.06
1-Butyl-3,5-dimethylpyridinium dicyanamide[75]	272.1	3.85	14.15
1-Butyl-3,5-dimethylpyridinium trifluoromethanesulfonate[75]	364.1	28.5	78.3
N-Butyronitrile pyridinium chloride[60]	342.4	13.9	40.6
N-Butyronitrile pyridinium tetrafluoroborate[60]	342.4	12.4	36.2
3,5-Dimethyl-1-octylpyridinium thiocyanate[75]	235.1	6.39	27.18
3,5-Dimethyl-1-octylpyridinium tetrafluoroborate[75]	329.1	28.07	85.29
3,5-Dimethyl-1-octylpyridinium iodide[75]	355.1	25.45	71.67
3,5-Dimethyl-1-octylpyridinium trifluoromethanesulfonate[75]	349.1	24.81	71.07
2,3,5-Trimethyl-1-octylpyridinium thiocyanate[75]	293.1	14.66	50.02
1-Butyl-2,3-dimethylpyridinium trifluoromethanesulfonate[75]	290.1	16.88	58.19
1-Butyl-2,3,5-trimethylpyridinium trifluoromethanesulfonate[75]	347.1	20.97	60.41
5-Ethyl-2-methyl-1-octylpyridinium iodide[75]	360.1	30.87	85.73
1-Hexyl-4-cyanopyridinium bis(trifluoromethylsulfonyl)imide[76]	280.2	18.83	67.20
3-Cyano-1-octylpyridinium bis(trifluoromethylsulfonyl)imide[76]	287.9	13.71	47.62
4-(1-Hexadecylheptadecyl)-1-methyl-pyridinium chloride[74]	337.1	56.9	168.8
1-Butyl-4-cyanopyridinium tricyanomethanide[77]	361.8	29.7	82.1
1-Decyloxymethyl-3-amido-pyridinium tetrafluoroborate[56]	361.9	51.26	141.64
1-(3-Cyanopropyl)pyridinium tricyanomethanide[78]	305.2	21.6	70.8
1-(Methoxymethyl)pyridinium bis(trifluoromethylsulfonyl)imide[62]	268.8	16.7	62.1
1-(2-Ethoxyethyl)pyridinium bis(trifluoromethylsulfonyl)imide[62]	259.2	19.5	75.2
1-(2-Propoxyethyl)pyridinium bis(trifluoromethylsulfonyl)imide[62]	256.4	3.3	12.9
Pyrrolidinium			
1,1-Dimethylpyrrolidinium thiocyanate[20]	368	9.97	27.09
1-Ethyl-1-methylpyrrolidinium bis(trifluoromethylsulfonyl)imide[79]	363.1	9.1	25.1
1-Methyl-1-propylpyrrolidinium thiocyanate[20]	280	23.3	83.3
1-Methyl-1-propylpyrrolidinium trifluoromethanesulfonate[77]	350.7	37.2	106.1
1-Methyl-1-propylpyrrolidinium bis(trifluoromethylsulfonyl)imide[80]	285.1	12.3	43.1
1-Butyl-1-methylpyrrolidinium bis(trifluoromethylsulfonyl)imide[81]	265.73	21.9	82.4
1-Butyl-1-methylpyrrolidinium tetracyanoborate[82]	295.3	35.6	120.6
1-Butyl-1-methylpyrrolidinium tricyanomethanide[83]	264.4	9.43	35.67
1-Butyl-1-methylpyrrolidinium trifluoromethanesulfonate[83]	272.9	12.07	44.23
1-Butyl-1-methylpyrrolidinium 1,1,2,2-tetrafluoroethanesulfonate[84]	318.5	10.9	34.2
1-Butyl-1-methylpyrrolidinium perfluorobutanesulfonate[85]	364	8.78	24.12
1-Pentyl-1-methylpyrrolidinium bis(trifluoromethylsulfonyl)imide[80]	281.1	22.5	80.0
1-Decyl-1-methylpyrrolidinium bis(trifluoromethylsulfonyl)imide[86]	283	8	28
1-Methyl-1-octadecylpyrrolidinium bis(trifluoromethylsulfonyl)imide[87]	345	32.3	94
1-Isobutyl-1-methylpyrrolidinium bis(trifluoromethylsulfonyl)imide[88]	272.9	13.1	48
1-(2-Methoxyethyl)-1-methylpyrrolidinium tris(pentafluoroethyl)trifluorophosphate[89]	273.3	14.3	52.3
Piperidinium			
1-Ethyl-1-methylpiperidinium bis(trifluoromethylsulfonyl)imide[90]	358.04	16.61	46.39
1-Methyl-1-propylpiperidinium bis(trifluoromethylsulfonyl)imide[91]	285.7	25.6	89.6
1-Butyl-1-methylpiperidinium trifluoromethanesulfonate[92]	309	23	75

Ammonium			
Methanammonium formate[93]	286.1	6.629	23.170
Trimethylammonium bis(trifluoromethylsulfonyl)imide[94]	357.4	17.36	48.58
Ethylammonium formate[93]	258.1	5.558	21.534
Ethylammonium acetate[93]	360.1	21.343	59.270
Ethylammonium nitrate[95]	285	13.2	46
Ethylammonium hydrogensulfate[93]	313.1	12.598	40.236
N,N-Diethyl-N-methylammonium methanesulfonate[92]	312	14	47
N,N-Diethyl-N-methylammonium trifluoromethanesulfonate[92]	268	17	63
Triethylammonium hydrogensulfate[96]	355.57	8.22	23.12
Propylammonium formate[93]	323.1	17.242	53.364
N,N-Dimethyl-N-propylammonium trifluoromethanesulfonate[97]	293.1	20	68
N-Ethyl-N,N-dimethyl-N-propylammonium bis(trifluoromethylsulfonyl)imide[80]	263.1	21.6	82.1
N,N-Diethyl-N-propylammonium trifluoromethanesulfonate[97]	259	21	81
N,N-Diethyl-N-methyl-N-propylammonium trifluoro(perfluoroethyl)borate[98]	327	9.58	29.3
N,N-Diethyl-N-methyl-N-propylammonium trifluoro(perfluoropropyl)borate[98]	330	5.18	15.7
N,N-Diethyl-N-methyl-N-propylammonium trifluoro(perfluorobutyl)borate[98]	327	5.85	17.9
N,N-Diethyl-N-methyl-N-propylammonium bis(trifluoromethylsulfonyl)imide[98]	287	4.53	15.8
N-Methyl-N,N-dipropylammonium trifluoromethanesulfonate[97]	290.1	14	48
Butylammonium formate[93]	275.1	7.984	29.022
N-Butyl-N-trimethylammonium bis(trifluoromethylsulfonyl)imide[33]	290.23	11.4	39.3
N-Butyl-N-ethyl-N,N-dimethylammonium ethylsulfate[99]	307	11.7	38.1
N-Butyl-N,N-diethyl-N-methylammonium trifluoro(perfluoroethyl)borate[98]	288	9.42	32.7
N-Butyl-N,N-diethyl-N-methylammonium trifluoro(perfluoropropyl)borate[98]	323	13.3	41.1
N-Butyl-N,N-diethyl-N-methylammonium trifluoro(perfluorobutyl)borate[98]	333	9.86	29.6
N-Butyl-N,N-diethyl-N-methylammonium bis(trifluoromethylsulfonyl)imide[98]	282	24.7	87.6
N-Butyl-N,N-dimethyl-N-propylammonium bis(trifluoromethylsulfonyl)imide[80]	293	16	53
Tetrabutylammonium chloride[100]	314.1	20.502	65.272
Pentylammonium formate[93]	285.1	7.858	27.562
Tetrapentylammonium thiocyanate[100]	322.65	19.665	60.948
Tetrahexylammonium nitrate[100]	345.15	17.573	50.914
Tetrahexylammonium tetrafluoroborate[100]	367	19.246	52.420
N-Heptyl-N,N,N-trihexylammonium nitrate[100]	345	33.5	97.1
N-Heptyl-N,N,N-trihexylammonium iodide[100]	371	20.502	55.239
N,N-Diheptyl-N,N-dihexylammonium iodide[100]	373	26.778	71.762
N,N,N-Triheptyl-N-heptanammonium bromide[100]	369	36	98
Trioctylpropylammonium bromide[100]	351	44.4	126.4
Tetraoctylammonium oleate[40]	252.4	19.4	76.9
N-Decyl-N,N,N-triethylammonium bis(trifluoromethylsulfonyl)imide[101]	264	19.21	72.76
N,N-Didecyl-N,N-dimethylammonium nitrate[102]	304.8	9.88	32.41
N-Dodecyl-N,N,N-triethylammonium bis(trifluoromethylsulfonyl)imide[101]	279.02	19.82	71.04
N,N,N-Triethyl-N-tetradecylammonium bis(trifluoromethylsulfonyl)imide[101]	295.6	33.58	119.29
Ethanolammonium nitrate[93]	324.1	12.41	38.29

Ethanolammonium methylsulfate[93]	372.1	26.671	71.677
Ethanolammonium tetrafluoroborate[45]	306.76	8.616	28.087
N-(2-Hydroxyethyl)-N,N,N-trimethylammonium butanesulfonate[99]	324	25	77
Choline tosylate[103]	346	19.1	51
N-Ethyl-N-(2-hydroxyethyl)-N,N-dimethylammonium dicyanamide[56]	282.7	8.6	30.4
N-Ethyl-N-(2-hydroxyethyl)-N,N-dimethylammonium methanesulfonate[99]	317	20.06	63.28
N-Ethyl-N-(2-hydroxyethyl)-N,N-dimethylammonium hexafluorophosphate[56]	272	10.6	39.0
N-Ethyl-N-(2-hydroxyethyl)-N,N-dimethylammonium butanesulfonate[99]	293	23.8	81.2
N-Ethyl-N-(2-hydroxyethyl)-N,N-dimethylammonium octanesulfonate[99]	320	29.9	93.4
N-(2-Hydroxyethyl)-N,N-dimethyl-N-propylammonium bromide[104]	372.6	4.12	11.06
N-(2-Hydroxyethyl)-N,N-dimethyl-N-undecyloxymethylammonium dicyanamide[105]	283.5	7.15	25.22
N-Hexyl-N-(2-hydroxyethyl)-N,N-dimethylammonium bromide[104]	355.3	3.78	10.64
Diethanolammonium tetrafluoroborate[45]	303.84	7.26	23.89
Tris(2-hydroxyethyl)ammonium tetrafluoroborate[45]	345.26	15.449	44.746
2-Methylpropylammonium formate[93]	299.1	6.196	20.715
3-Methylbutylammonium formate[93]	320.1	12.52	39.11
2-Methylbutylammonium formate[93]	272.1	6.926	25.454
(2-Decanoyloxyethyl)dimethylpentyloxymethylammonium trifluoroacetate[105]	279.8	7.83	27.98
Choline bis(trifluoromethylsulfonyl)imide[106]	295.2	4.68	15.85
2-Methoxyethyl-N,N,N-trimethylammonium tetrafluoroborate[98]	327	15.0	45.8
2-Methoxyethyl-N,N,N-trimethylammonium trifluoro(trifluoromethyl)borate[98]	350	11.7	33.3
2-Methoxyethyl-N,N,N-trimethylammonium trifluoro(perfluoroethyl)borate[98]	303	11.6	38.4
2-Methoxyethyl-N,N,N-trimethylammonium trifluoro(perfluoropropyl)borate[98]	296	13.6	45.9
2-Methoxyethyl-N,N,N-trimethylammonium trifluoro(perfluorobutyl)borate[98]	323	17.6	54.4
2-Methoxyethyl-N,N,N-trimethylammonium bis(trifluoromethylsulfonyl)imide[98]	310	26.5	85.6
N-Ethyl-2-methoxyethyl-N,N-dimethylammonium trifluoro(trifluoromethyl)borate[98]	281	8.1	28.8
N-Ethyl-2-methoxyethyl-N,N-dimethylammonium tetrafluoroborate[98]	277	15.2	55.0
N-Ethyl-2-methoxyethyl-N,N-dimethylammonium trifluoro(perfluoroethyl)borate[98]	240	13.9	57.9
N-Ethyl-2-methoxyethyl-N,N-dimethylammonium trifluoro(perfluorobutyl)borate[98]	245	14.7	60.2
N,N-Diethyl-2-methoxy-N-methylethan-1-ammonium tetrafluoroborate[98]	281	17.1	61.0
N,N-Diethyl-2-methoxyethyl-N-methylammonium trifluoro(trifluoromethyl)borate[98]	251	7.5	29.9
N,N,N-Triethyl-2-methoxyethylammonium trifluoro(trifluoromethyl)borate[98]	283	17.2	60.7
N,N,N-Triethyl-2-methoxyethylammonium trifluoro(perfluoroethyl)borate[98]	276	24.4	88.4
N,N,N-Triethyl-2-methoxyethylammonium trifluoro(perfluoropropyl)borate[98]	279	8.6	30.7
N,N,N-Triethyl-2-methoxyethylammonium trifluoro(perfluorobutyl)borate[98]	284	7.4	26.1
N,N,N-Triethyl-2-methoxyethylammonium tetrafluoroborate[98]	329	18.8	57.0
N,N,N-Triethyl-2-methoxyethylammonium	293	23.8	81.3

bis(trifluoromethylsulfonyl)imide[98]			
N,N-Diallyl-N-methylammonium trifluoromethanesulfonate[97]	254	11	43
N-Allyl-N,N-dimethylammonium trifluoromethanesulfonate[97]	289	21	73
N-Allyl-N,N-diethylammonium trifluoromethanesulfonate[97]	259	21	81
N-Butyronitrile-N,N,N-trimethylammonium tetrafluoroborate[60]	334.1	12.3	36.8
N-Butyronitrile-N,N,N-trimethylammonium bis(trifluoromethylsulfonyl)imide[60]	331.3	20.2	61.0
N,N-Dimethyl-N-isopropyl-N-propylammonium bis(trifluoromethylsulfonyl)imide[86]	290	14	48
N-Butyl-N,N-dimethyl-N-isopropylammonium bis(trifluoromethylsulfonyl)imide[86]	283	10	35
N-Decyl-N-isopropyl-N,N-dimethylammonium bis(trifluoromethylsulfonyl)imide[86]	270	26	96
Phosphonium			
Tetrabutylphosphonium methanesulfonate[107]	335.35	11.105	33.115
Tetrabutylphosphonium tris(pentafluoroethyl)trifluorophosphate[52]	347	7.8	22.5
Tetraoctylphosphonium bis(trifluoromethylsulfonyl)imide[108]	284.3	45.43	159.80
Other			
N-Octylbenzothiazolium hexafluorophosphate[109]	334.22	15	45
3-Heptylbenzo[d]thiazolium hexafluorophosphate[109]	359.3	20.1	55.9
N-Hexylbenzothiazolium hexafluorophosphate[109]	358.79	23.5	65.5
1,5-Diamino-4-methyltetrazolium dinitramide[110]	358	26.1	72.9
1-Butylquinolinium bis(trifluoromethylsulfonyl)imide[111]	329.62	44.14	133.91
2-Butylisoquinolinium bis(trifluoromethylsulfonyl)imide[112]	321	46.13	143.71
1-Hexylisoquinolinium bis(trifluoromethylsulfonyl)imide[113]	327.2	58.64	179.22
1-Hexylquinolinium bis(trifluoromethylsulfonyl)imide[114]	317.2	63.54	200.32
1-Octylquinolinium bis(trifluoromethylsulfonyl)imide[115]	321.3	62.91	195.80
Trimethylsulfonium bis(trifluoromethylsulfonyl)imide[94]	357.4	53	148
Diethylmethylsulfonium bis(trifluoromethylsulfonyl)imide[116]	256.5	12.5	48.7
Triethylsulfonium bis(trifluoromethylsulfonyl)imide[71]	262.8	6.98	26.56
Hexyloctamethylferrocenium tetracyanoethylene[117]	354.1	39	110
Butyloctamethylferrocenium bis(trifluoromethylsulfonyl)imide[117]	307.6	26.54	86.3
Hexyloctamethylferrocenium bis(trifluoromethylsulfonyl)imide[117]	300.9	25.45	84.6
1-Hexyl-1,4-diaza[2.2.2]bicyclooctanium bis(trifluoromethylsulfonyl)imide[118]	309	5.3	17.0
4-(3-Cyanopropyl)-4-methylmorpholinium tricyanomethanide[78]	325.5	21.8	67.0
Tetramethylguanidinium nitrate[119]	368.6	19.84	53.83
Pyrimethanil laurate[120]	321.52	67.245	209.28
N-Benzyl-N-dimethyl-Ntetradecylammonium vannilliate[121]	320.5	35.59	111.05
Lead dibutanoate[122]	346.5	14.7	42.4

Table S2. T_m, $\Delta_{fus}H$, and $\Delta_{fus}S$ data for alkali halide.[123]

Alkali halide	T_m / K	$\Delta_{fus}H$ / kJ mol^{-1}	$\Delta_{fus}S$ / J K^{-1} mol^{-1}
LiF	1121	27.1	24.1
LiCl	883	19.9	22.6
LiBr	823	17.7	21.5
LiI	742	14.6	19.7
NaF	1268	33.6	25.9
NaCl	1073	28.0	26.1
NaBr	1020	26.1	25.6
NaI	933	23.6	25.3
KF	1131	28.2	25.0
KCl	1043	26.5	25.4
KBr	1007	25.5	25.4
KI	954	24.0	25.2
RbF	1068	25.7	24.1
RbCl	995	23.7	23.8
RbBr	965	23.3	24.1
RbI	920	22.0	24.0
CsF	986	21.7	22.3
CsCl	918	20.3	22.0
CsBr	909	23.6	25.9
CsI	899	23.6	26.2

Table S3. T_m, $\Delta_{fus}H$, and $\Delta_{fus}S$ data for $[C_1mim]X$.[a]

IL	T_m / K	$\Delta_{fus}H$ / kJ mol^{-1}	$\Delta_{fus}S$ / J K^{-1} mol^{-1}
$[C_1mim]I$	361.4	12.3	34.1
$[C_1mim]NO_3$	337.2	19.8	58.8
$[C_1mim]CH_3CO_2$	308.7	14.2	46.0
$[C_1mim]CF_3CO_2$	326.7	19.0	58.1
$[C_1mim]CH_3SO_3$	367.4	23.1	62.9
$[C_1mim]CF_3SO_3$	310.5	18.5	59.7
$[C_1mim][OTs]$	365.2	24.6	67.4
$[C_1mim]SCN$	295.2	15.7	53.2
$[C_1mim]N(CN)_2$	306.4	15.4	50.3
$[C_1mim]C(CN)_3$	322.6	21.7	67.4
$[C_1mim]PF_6$[b] [124]	364.3	17.3	47.6
$[C_1mim]CH_3SO_4$[c] [17]	308.9	16.58	53.67

[a]Melting point (T_m) was taken from the onset temperature from the DSC traces. Standard uncertainties are $u(T_m)$ = 0.8 K; $u(\Delta_{fus}H)$ = 0.4 kJ mol^{-1}; $u(\Delta_{fus}S)$ = 1.2 J K^{-1} mol^{-1}. [b]The data is not contained in ILThermo. [c]The data is contained in ILThermo as listed in Table S1.

Table S4. Solid-solid phase transition temperature ($T_{s\text{-}s}$), enthalpy change ($\Delta_{s\text{-}s}H$), and entropy change ($\Delta_{s\text{-}s}S$) during heating for [C_1mim]X. A slight exothermic peak in [C_1mim]SCN (Figure S1H) is omitted because phase transitions do not occur exothermically during heating.

IL	$T_{s\text{-}s}$ / K	$\Delta_{s\text{-}s}H$ / kJ mol^{-1}	$\Delta_{s\text{-}s}S$ / J K^{-1} mol^{-1}
[C_1mim]NO_3	274.1	0.5	1.9
[C_1mim][OTs]	325.3	2.0	6.2
[C_1mim]$N(CN)_2$	252.3	1.0	4.0
[C_1mim]$N(CN)_2$	265.2	2.3	8.7

Table S5. Calculated gas-phase entropies (J K^{-1} mol^{-1}) at 1 bar for NaCl and the ILs.

Ion/ion pair	S_{tra}	S_{rot}	S_{vib}	Total
298.15 K				
Single ion				
Na^+	147.8	0.0	0.0	147.8
Cl^-	153.1	0.0	0.0	153.1
$[C_2mim]^+$	167.5	117.8	93.8	379.1
$[C_4mim]^+$	170.3	126.2	148.0	444.4
PF_6^-	170.8	88.6	45.3	304.7
Ion pair				
NaCl	159.4	65.5	4.9	229.7
$[C_2mim]PF_6$	177.9	134.9	234.2	547.0
$[C_4mim]PF_6$	179.2	139.0	286.2	604.4
Melting point[a]				
Single ion				
Na^+	174.8	0.0	0.0	174.8
Cl^-	180.0	0.0	0.0	180.0
$[C_2mim]^+$	170.1	119.4	107.1	396.6
PF_6^-	173.4	90.1	55.2	318.7
$[C_4mim]^+$	168.6	125.1	135.2	428.9
PF_6^-	169.1	87.5	39.5	296.1
Ion pair				
NaCl	186.3	76.3	14.8	277.4
$[C_2mim]PF_6$	180.5	136.5	263.8	580.8
$[C_4mim]PF_6$	177.5	138.0	265.6	581.1

[a]Estimated from the MD simulations in this work (NaCl: 1089K, $[C_2mim]PF_6$: 338 K, and $[C_4mim]PF_6$: 275 K).

Table S6. Results from the PSCP cycle. All units are in kJ mol^{-1}. Reference temperatures of NaCl, [C_2mim]PF_6, and [C_4mim]PF_6 were 1100 K, 380 K, and 340 K, respectively.

	$\Delta_1 A$	$\Delta_2 A$	$\Delta_3 A$	$\Delta_4 A$	$p\Delta V$	$\Delta_{ref} G$
NaCl	574.75 ± 0.02	109.45 ± 0.01	−13.06 ± 0.00	−671.42 ± 0.01	0.0009	−0.29 ± 0.02
[C_2mim]PF_6	134.55 ± 0.01	85.83 ± 0.02	−5.93 ± 0.00	−216.79 ± 0.02	0.0015	−2.33 ± 0.04
[C_4mim]PF_6	144.61 ± 0.03	117.83 ± 0.02	−7.25 ± 0.00	−259.59 ± 0.02	0.0018	−4.40 ± 0.03

Table S7. Calculated T_m, $\Delta_{fus}H$, and $\Delta_{fus}S$ values of NaCl and the ILs. The reported experimental and MD values are also shown.

Salt	MD (This work)	Exp.	MD (reported)
T_m / K			
NaCl	1088.8 ± 0.9	1073[123]	1082[125]
$[C_2mim]PF_6$	337.7 ± 0.6	332.80[126], 334.1[127], 334.2[128]	330[5, 6], 330[7], 355[4]
$[C_4mim]PF_6$	275.4 ± 0.4	283.51[35], 280.03[129], 281.83[130], 282.3[43]	284[5, 6], 284[7]
$\Delta_{fus}H$ / kJ mol^{-1}			
NaCl	28.13 ± 0.01	28.0[123]	28.1[125]
$[C_2mim]PF_6$	18.17 ± 0.05	17.86[126], 17.99[127], 17.7[128]	17.70[5, 6], 17.32[7], 19.3[4]
$[C_4mim]PF_6$	17.95 ± 0.13	19.601[35], 19.91[129], 20.67[130], 20.9[43]	18.83[5, 6], 17.95[7]
$\Delta_{fus}S$ / J K^{-1} mol^{-1}			
NaCl	25.83 ± 0.02	26.1[123]	25.9[125]
$[C_2mim]PF_6$	53.81 ± 0.17	53.67[126], 53.85[127], 53.2[128]	53.64[5, 6], 52.51[7], 51.6[4]
$[C_4mim]PF_6$	65.16 ± 0.37	69.14[35], 71.10[129], 73.34[130], 73.5[43]	66.32[5, 6], 63.22[7]

Table S8. Populations of conformations and S_{confor} for $[C_2mim]PF_6$ at 338 K.

	Crystal	Liquid
p	0.010 ± 0.000	0.109 ± 0.000
n	0.973 ± 0.001	0.445 ± 0.002
n’	0.017 ± 0.001	0.446 ± 0.002
S_{confor} / J K^{-1} mol^{-1}	1.17 ± 0.02	8.00 ± 0.00

Table S9. Populations of conformations and S_{confor} for $[C_4mim]PF_6$ at 275 K

	Crystal	Liquid
ptt	0	0.022 ± 0.001
ptg	0	0.008 ± 0.000
ptg'	0	0.007 ± 0.000
pgt	0	0.003 ± 0.001
pgg	0	0.001 ± 0.000
pgg'	0	0.000 ± 0.000
pg't	0	0.003 ± 0.000
pg'g	0	0.000 ± 0.000
pg'g'	0	0.001 ± 0.000
ntt	0.015 ± 0.001	0.188 ± 0.006
ntg	0.000 ± 0.000	0.069 ± 0.003
ntg'	0.000 ± 0.000	0.056 ± 0.002
ngt	0.094 ± 0.009	0.054 ± 0.004
ngg	0.001 ± 0.001	0.019 ± 0.001
ngg'	0.000 ± 0.000	0.002 ± 0.000
ng't	0.888 ± 0.010	0.061 ± 0.004
ng'g	0.000 ± 0.000	0.002 ± 0.000
ng'g'	0.001 ± 0.000	0.026 ± 0.002
ntt	0.000 ± 0.000	0.186 ± 0.005
ntg	0.000 ± 0.000	0.061 ± 0.003
ntg'	0.000 ± 0.000	0.072 ± 0.004
ngt	0.000 ± 0.000	0.056 ± 0.001
ngg	0.000 ± 0.000	0.021 ± 0.004
ngg'	0.000 ± 0.000	0.002 ± 0.001
ng't	0.000 ± 0.001	0.057 ± 0.004
ng'g	0.000 ± 0.000	0.002 ± 0.000
ng'g'	0.000 ± 0.000	0.021 ± 0.001
S_{confor} / J K^{-1} mol^{-1}	3.43 ± 0.19	21.40 ± 0.11

Table S10. Absolute entropies (J K^{-1} mol^{-1}) of the salts in the crystal state calculated by the MD simulations (this work) and reported experimentally. Configurational entropies (S_{config}) in the crystal state are assumed to be zero, which would be reasonable because the positional and orientational disorders of the ILs were negligible, judging from the MD trajectories.

		MD (This work)					Exp.
		S_{tra}	S_{rot}	S_{vib}	S_{confor}	Total	
NaCl at 1089 K	Na^+	67.5 ± 0.0	0	0	0	140.3 ± 0.0	145.0 [131]
	Cl^-	72.8 ± 0.0	0	0	0		
[C_2mim]PF_6 at 338 K	[C_2mim]$^+$	61.7 ± 0.1	55.8 ± 0.1	111.6 ± 0.3	1.17 ± 0.02	440.9 ± 0.7	n/a
	PF_6^-	69.1 ± 0.1	73.5 ± 0.1	68.0 ± 0.2	0		
[C_4mim]PF_6 at 275 K	[C_4mim]$^+$	56.3 ± 0.1	46.7 ± 0.2	126.2 ± 0.2	3.43 ± 0.19	411.1 ± 0.8	392.9 [35]
	PF_6^-	62.8 ± 0.2	63.7 ± 0.1	51.9 ± 0.1	0		

Table S11. Decompositions of calculated $\Delta_{fus}S$ of NaCl, [C_2mim]PF_6, and [C_4mim]PF_6. Unit is in J K^{-1} mol^{-1}.

		$\Delta_{kin}S$				$\Delta_{str}S$			$\Delta_{fus}S$
		$\Delta_{tra}S$	$\Delta_{rot}S$	$\Delta_{vib}S$	Total	$\Delta_{confor}S$	$\Delta_{config}S$	Total	
NaCl at 1089 K	Na^+	11.7 ± 0.0	0.0	0.0	25.2 ± 0.1	0	0.7 ± 0.1	0.7 ± 0.1	25.8 ± 0.0
	Cl^-	13.4 ± 0.0	0.0	0.0		0			
[C_2mim]PF_6 at 338 K	[C_2mim]$^+$	4.1 ± 0.2	7.0 ± 0.1	−0.2 ± 0.3	16.9 ± 0.9	6.8 ± 0.0	30.0 ± 0.9	36.9 ± 0.9	53.8 ± 0.2
	PF_6^-	4.3 ± 0.2	2.1 ± 0.2	−0.4 ± 0.2		0			
[C_4mim]PF_6 at 275 K	[C_4mim]$^+$	3.8 ± 0.2	9.3 ± 0.2	−1.2 ± 0.2	19.1 ± 1.2	18.0 ± 0.2	28.1 ± 1.3	46.1 ± 1.3	65.2 ± 0.4
	PF_6^-	3.4 ± 0.3	5.6 ± 0.4	−1.9 ± 0.1		0			

Table S12. Diffusion coefficients of ions in NaCl (1089 K), $[C_2mim]PF_6$ (338 K), and $[C_4mim]PF_6$ (275K) in addition to their experimental viscosity data.

Salt	Ion	Simulated D (this work) / $m^2\ s^{-1}$	Experimental D / $m^2\ s^{-1}$	Experimental viscosity / mPa s
NaCl	Na^+	$8.44 \pm 0.24 \times 10^{-9}$	7.75×10^{-9} [132]	1[133]
	Cl^-	$7.75 \pm 0.26 \times 10^{-9}$	6.13×10^{-9} [132]	
$[C_2mim]PF_6$	$[C_2mim]^+$	$6.13 \pm 0.27 \times 10^{-11}$	n/a	28[134]
	PF_6^-	$4.00 \pm 0.17 \times 10^{-11}$	n/a	
$[C_4mim]PF_6$	$[C_4mim]^+$	$1.19 \pm 0.16 \times 10^{-12}$	1.18×10^{-12} [135]	1459[136]
	PF_6^-	$0.50 \pm 0.07 \times 10^{-12}$	0.82×10^{-12} [135]	

SI References

1. S. T. Lin, M. Blanco and W. A. Goddard Iii, *J. Chem. Phys.*, 2003, **119**, 11792-11805.
2. S. T. Lin, P. K. Maiti and W. A. Goddard Iii, *J. Phys. Chem. B*, 2010, **114**, 8191-8198.
3. D. M. Eike, J. F. Brennecke and E. J. Maginn, *J. Chem. Phys.*, 2005, **122**, 014115.
4. K. Bernardino, Y. Zhang, M. C. C. Ribeiro and E. J. Maginn, *J. Chem. Phys.*, 2020, **153**, 044504.
5. Y. Zhang and E. J. Maginn, *J. Phys. Chem. B*, 2012, **116**, 10036-10048.
6. Y. Zhang and E. J. Maginn, *Phys. Chem. Chem. Phys.*, 2012, **14**, 12157-12164.
7. Y. Zhang and E. J. Maginn, *Phys. Chem. Chem. Phys.*, 2014, **16**, 13489-13499.
8. E. A. Turner, C. C. Pye and R. D. Singer, *J. Phys. Chem. A*, 2003, **107**, 2277-2288.
9. Y. Umebayashi, T. Fujimori, T. Sukizaki, M. Asada, K. Fujii, R. Kanzaki and S.-I. Ishiguro, *J. Phys. Chem. A*, 2005, **109**, 8976-8982.
10. R. Ozawa, S. Hayashi, S. Saha, A. Kobayashi and H.-o. Hamaguchi, *Chem. Lett.*, 2003, **32**, 948-949.
11. S.-T. Lin, M. Blanco and W. A. Goddard, *J. Chem. Phys.*, 2003, **119**, 11792-11805.
12. T. A. Pascal, D. Schärf, Y. Jung and T. D. Kühne, *J. Chem. Phys.*, 2012, **137**, 244507.
13. M. A. Caro, T. Laurila and O. Lopez-Acevedo, *J. Chem. Phys.*, 2016, **145**, 244504.
14. Q. Dong, C. D. Muzny, A. Kazakov, V. Diky, J. W. Magee, J. A. Widegren, R. D. Chirico, K. N. Marsh and M. Frenkel, *J. Chem. Eng. Data*, 2007, **52**, 1151-1159.
15. A. F. Kazakov, J. W. Magee, R. D. Chirico, V. Diky, K. G. Kroenlein, C. D. Muzny and M. D. Frenkel, *Journal*, 2021.
16. V. N. Emel'yanenko, S. P. Verevkin, A. Heintz, K. Voss and A. Schulz, *J. Phys. Chem. B*, 2009, **113**, 9871-9876.
17. U. Domańska, A. Pobudkowska and F. Eckert, *Green Chem.*, 2006, **8**, 268-276.
18. H. Tokuda, K. Hayamizu, K. Ishii, M. A. B. H. Susan and M. Watanabe, *J. Phys. Chem. B*, 2005, **109**, 6103-6110.
19. P. Keil and A. König, *Thermochim. Acta*, 2011, **524**, 202-204.
20. J. M. Pringle, J. Golding, C. M. Forsyth, G. B. Deacon, M. Forsyth and D. R. MacFarlane, *J. Mater. Chem.*, 2002, **12**, 3475-3480.
21. S. Zarei, S. Abdolrahimi and G. Pazuki, *Fluid Phase Equilib.*, 2019, **497**, 140-150.
22. V. Štejfa, J. Rohlíček and C. Červinka, *J. Chem. Thermodyn.*, 2020, **142**, 106020.
23. Y. U. Paulechka, G. J. Kabo, A. V. Blokhin, A. S. Shaplov, E. I. Lozinskaya and Y. S. Vygodskii, *J. Chem. Thermodyn.*, 2007, **39**, 158-166.
24. M. E. V. Valkenburg, R. L. Vaughn, M. Williams and J. S. Wilkes, *Thermochim. Acta*, 2005, **425**, 181-188.
25. H. a. Every, A. G. Bishop, D. R. MacFarlane, G. Orädd and M. Forsyth, *J. Mater. Chem.*, 2001, **11**, 3031-3036.
26. P. B. P. Serra, F. M. S. Ribeiro, M. A. A. Rocha, M. Fulem, K. Růžička, J. A. P. Coutinho and L. M. Santos, *J. Mol. Liq.*, 2017, **248**, 678-687.
27. Y. U. Paulechka, A. V. Blokhin, G. J. Kabo and A. A. Strechan, *J. Chem. Thermodyn.*, 2007, **39**, 866-877.

28. E. Vataščin and V. Dohnal, *J. Chem. Thermodyn.*, 2018, **119**, 114-126.
29. K. Matsumoto, E. Nishiwaki, T. Hosokawa, S. Tawa, T. Nohira and R. Hagiwara, *J. Phys. Chem. C*, 2017, **121**, 9209-9219.
30. Y. U. Paulechka and A. V. Blokhin, *J. Chem. Thermodyn.*, 2014, **79**, 94-99.
31. A. Efimova, G. Hubrig and P. Schmidt, *Thermochim. Acta*, 2013, **573**, 162-169.
32. A. A. Strechan, A. G. Kabo, Y. U. Paulechka, A. V. Blokhin, G. J. Kabo, A. S. Shaplov and E. I. Lozinskaya, *Thermochim. Acta*, 2008, **474**, 25-31.
33. Y. U. Paulechka, A. G. Kabo, A. V. Blokhin, G. J. Kabo and M. P. Shevelyova, *J. Chem. Eng. Data*, 2010, **55**, 2719-2724.
34. A. A. Strechan, Y. U. Paulechka, A. V. Blokhin and G. J. Kabo, *J. Chem. Thermodyn.*, 2008, **40**, 632-639.
35. G. J. Kabo, A. V. Blokhin, Y. U. Paulechka, A. G. Kabo, M. P. Shymanovich and J. W. Magee, *J. Chem. Eng. Data*, 2004, **49**, 453-461.
36. R. A. M. Faria, T. F. M. Vieira, C. I. Melo and E. Bogel-ŁUkasik, *J. Chem. Eng. Data*, 2016, **61**, 3116-3126.
37. A. A. Strechan, Y. U. Paulechka, A. G. Kabo, A. V. Blokhin and G. J. Kabo, *J. Chem. Eng. Data*, 2007, **52**, 1791-1799.
38. P. Wasserscheid, R. van Hal and A. Bösmann, *Green Chem.*, 2002, **4**, 400-404.
39. Y. Shimizu, Y. Ohte, Y. Yamamura and K. Saito, *Chem. Lett.*, 2007, **36**, 1484-1485.
40. B. Monteiro, L. Maria, A. Cruz, J. M. Carretas, J. Marçalo and J. P. Leal, *Thermochim. Acta*, 2020, **684**, 178482.
41. D. G. Archer, *NISTIR No. 6645.*, 2006.
42. Y. U. Paulechka, A. V. Blokhin and G. J. Kabo, *Thermochim. Acta*, 2015, **604**, 122-128.
43. F. Nemoto, M. Kofu and O. Yamamuro, *J. Phys. Chem. B*, 2015, **119**, 5028-5034.
44. U. Domańska and E. Bogel-Łukasik, *Ind. Eng. Chem. Res.*, 2003, **42**, 6986-6992.
45. J. Zhu, L. Bai, B. Chen and W. Fei, *Chem. Eng. J.*, 2009, **147**, 58-62.
46. E. Paulechka, A. V. Blokhin, A. S. M. C. Rodrigues, M. A. A. Rocha and L. M. Santos, *J. Chem. Thermodyn.*, 2016, **97**, 331-340.
47. C. A. S. Trindade, Z. P. Visak, R. Bogel-Łukasik, E. Bogel-Łukasik and M. N. d. Ponte, *Ind. Eng. Chem. Res.*, 2010, **49**, 4850-4857.
48. U. Domańska and M. Wlazło, *J. Chem. Thermodyn.*, 2016, **103**, 76-85.
49. H. Weiss, J. Mars, H. Li, G. Kircher, O. Ivanova, A. Feoktystov, O. Soltwedel, M. Bier and M. Mezger, *J. Phys. Chem. B*, 2017, **121**, 620-629.
50. T. Endo, K. Sakaguchi, K. Higashihara and Y. Kimura, *J. Chem. Eng. Data*, 2019, **64**, 5857-5868.
51. U. Domanska, A. Rekawek and A. Marciniak, *J. Chem. Eng. Data*, 2008, **53**, 1126-1132.
52. J. Salgado, T. Teijeira, J. J. Parajó, J. Fernández and J. Troncoso, *J. Chem. Thermodyn.*, 2018, **123**, 107-116.
53. P. B. P. Serra, F. M. S. Ribeiro, M. A. A. Rocha, M. Fulem, K. Růžička and L. M. Santos, *J. Chem. Thermodyn.*, 2016, **100**, 124-130.
54. S. V. Dzyuba and R. A. Bartsch, *ChemPhysChem*, 2002, **3**, 161-166.
55. R. Tao, G. Tamas, L. Xue, S. L. Simon and E. L. Quitevis, *J. Chem. Eng. Data*, 2014, **59**, 2717-2724.

56. U. Domańska, *Thermochim. Acta*, 2006, **448**, 19-30.
57. L. Xue, E. Gurung, G. Tamas, Y. P. Koh, M. Shadeck, S. L. Simon, M. Maroncelli and E. L. Quitevis, *J. Chem. Eng. Data*, 2016, **61**, 1078-1091.
58. U. Domańska and A. Marciniak, *Fluid Phase Equilib.*, 2007, **260**, 9-18.
59. M. Wlazło, M. Zawadzki and U. Domańska, *J. Chem. Thermodyn.*, 2018, **116**, 316-322.
60. Q. Zhang, Z. Li, J. Zhang, S. Zhang, L. Zhu, J. Yang, X. Zhang and Y. Deng, *J. Phys. Chem. B*, 2007, **111**, 2864-2872.
61. D.-W. Fang, L. Gong, X.-T. Fan, K.-H. Liang, X.-X. Ma and J. Wei, *J. Therm. Anal. Calorim.*, 2019, **138**, 1437-1442.
62. Z. Chen, Y. Huo, J. Cao, L. Xu and S. Zhang, *Ind. Eng. Chem. Res.*, 2016, **55**, 11589-11596.
63. Q.-S. Liu, M. Yang, P.-F. Yan, X.-M. Liu, Z.-C. Tan and U. Welz-Biermann, *J. Chem. Eng. Data*, 2010, **55**, 4928-4930.
64. M. García-Andreu, M. Castro, I. Gascón and C. Lafuente, *J. Chem. Thermodyn.*, 2016, **103**, 395-402.
65. Q.-S. Liu, Z.-C. Tan, U. Welz-Biermann and X.-X. Liu, *J. Chem. Thermodyn.*, 2014, **68**, 82-89.
66. B. Tong, Q.-S. Liu, Z.-C. Tan and U. Welz-Biermann, *J. Phys. Chem. A*, 2010, **114**, 3782-3787.
67. I. Bandres, F. M. Royo, I. Gascón, M. Castro and C. Lafuente, *J. Phys. Chem. B*, 2010, **114**, 3601-3607.
68. C. M. S. S. Neves, M. L. S. Batista, A. F. M. Cláudio, L. M. N. B. F. Santos, I. M. Marrucho, M. G. Freire and J. A. P. Coutinho, *J. Chem. Eng. Data*, 2010, **55**, 5065-5073.
69. T. M. Letcher, D. Ramjugernath, K. Tumba, M. Królikowski and U. Domańska, *Fluid Phase Equilib.*, 2010, **294**, 89-97.
70. U. Domanska, M. Królikowski, A. Pobudkowska and T. M. Letcher, *J. Chem. Eng. Data*, 2009, **54**, 1435-1441.
71. U. Domańska, M. Królikowski and K. Ślesińska, *J. Chem. Thermodyn.*, 2009, **41**, 1303-1311.
72. A. B. Pereiro, A. Rodriguez, M. Blesic, K. Shimizu, J. N. Canongia Lopes and L. P. N. Rebelo, *J. Chem. Eng. Data*, 2011, **56**, 4356-4363.
73. U. Domańska, M. Królikowski, A. Pobudkowska and P. Bocheńska, *J. Chem. Thermodyn.*, 2012, **55**, 225-233.
74. E. J. R. Sudholter, J. B. F. N. Engberts and W. H. De Jeu, *J. Phys. Chem.*, 1982, **86**, 1908-1913.
75. N. Papaiconomou, J. Estager, Y. Traore, P. Bauduin, C. Bas, S. Legeai, S. Viboud and M. Draye, *J. Chem. Eng. Data*, 2010, **55**, 1971-1979.
76. U. Domańska, K. Skiba, M. Zawadzki, K. Paduszyński and M. Królikowski, *J. Chem. Thermodyn.*, 2013, **56**, 153-161.
77. U. Domańska, M. Roguszewska, M. Królikowski, D. Ramjugernath and P. Naidoo, *J. Chem. Thermodyn.*, 2015, **83**, 90-96.
78. M. Królikowski, M. Więckowski and M. Zawadzki, *J. Chem. Thermodyn.*, 2020, **149**, 106149.
79. A. J. Hill, J. Huang, J. Efthimiadis, P. Meakin, M. Forsyth and D. R. Macfarlane,

Solid State Ionics, 2002, **154**, 119-124.
80. D. R. Macfarlane, P. Meakin, N. Amini and M. Forsyth, *J. Phys.: Condens. Matter*, 2001, **13**, 8257.
81. Y. Shimizu, Y. Ohte, Y. Yamamura, S. Tsuzuki and K. Saito, *J. Phys. Chem. B*, 2012, **116**, 5406-5413.
82. U. Domańska, M. Królikowski and W. E. Acree Jr, *J. Chem. Thermodyn.*, 2011, **43**, 1810-1817.
83. U. Domańska, P. Okuniewska and A. Markowska, *Fluid Phase Equilib.*, 2016, **424**, 68-78.
84. M. Havlová and V. Dohnal, *J. Chem. Thermodyn.*, 2018, **121**, 129-144.
85. M. L. Ferreira, M. J. Pastoriza-Gallego, J. M. M. Araujo, J. N. Canongia Lopes, L. P. N. Rebelo, M. M. Piñeiro, K. Shimizu and A. B. Pereiro, *J. Phys. Chem. C*, 2017, **121**, 5415-5427.
86. H. Jin, B. O'Hare, J. Dong, S. Arzhantsev, G. a. Baker, J. F. Wishart, A. J. Benesi and M. Maroncelli, *J. Phys. Chem. B*, 2008, **112**, 81-92.
87. K. Goossens, K. Lava, P. Nockemann, K. Van Hecke, L. Van Meervelt, K. Driesen, C. Görller-Walrand, K. Binnemans and T. Cardinaels, *Chem. Eur. J.*, 2009, **15**, 656-674.
88. A. S. M. C. Rodrigues, H. F. D. Almeida, M. G. Freire, J. A. Lopes-da-Silva, J. A. P. Coutinho and L. M. Santos, *Fluid Phase Equilib.*, 2016, **423**, 190-202.
89. A. Marciniak and M. Królikowski, *J. Chem. Thermodyn.*, 2012, **49**, 154-158.
90. U. Domańska, M. Krolikowska and K. Paduszyński, *Fluid Phase Equilib.*, 2011, **303**, 1-9.
91. K. Paduszyński, J. Chiyen, D. Ramjugernath, T. M. Letcher and U. Domańska, *Fluid Phase Equilib.*, 2011, **305**, 43-52.
92. J. J. Parajó, M. Villanueva, P. B. Sánchez and J. Salgado, *J. Chem. Thermodyn.*, 2018, **126**, 1-10.
93. T. L. Greaves, A. Weerawardena, C. Fong, I. Krodkiewska and C. J. Drummond, *J. Phys. Chem. B*, 2006, **110**, 22479-22487.
94. E. Couadou, J. Jacquemin, H. Galiano, C. Hardacre and M. Anouti, *J. Phys. Chem. B*, 2013, **117**, 1389-1402.
95. J. Salgado, J. J. Parajó, M. Villanueva, J. R. Rodríguez, O. Cabeza and L. M. Varela, *J. Chem. Thermodyn.*, 2019, **134**, 164-174.
96. L. E. Shmukler, M. S. Gruzdev, N. O. Kudryakova, Y. A. Fadeeva, A. M. Kolker and L. P. Safonova, *J. Mol. Liq.*, 2018, **266**, 139-146.
97. T. Yasuda, H. Kinoshita, M. S. Miran, S. Tsuzuki and M. Watanabe, *J. Chem. Eng. Data*, 2013, **58**, 2724-2732.
98. Z. B. Zhou, H. Matsumoto and K. Tatsumi, *Chem. Eur. J.*, 2005, **11**, 752-766.
99. M. Mahrova, M. Vilas, A. Dominguez, E. Gomez, N. Calvar and E. Tojo, *J. Chem. Eng. Data*, 2012, **57**, 241-248.
100. T. G. Coker, J. Ambrose and G. J. Janz, *J. Am. Chem. Soc.*, 1970, **92**, 5293-5297.
101. K. Machanová, Z. Wagner, A. Andresová, J. Rotrekl, A. Boisset, J. Jacquemin and M. Bendová, *J. Solution Chem.*, 2015, **44**, 790-810.
102. U. Domańska, K. Ługowska and J. Pernak, *J. Chem. Thermodyn.*, 2007, **39**, 729-736.
103. J. J. Parajo, M. Villanueva, J. Troncoso and J. Salgado, *J. Chem. Thermodyn.*,

2020, **141**, 105947.
104. U. Domańska and R. Bogel-Łukasik, *J. Phys. Chem. B*, 2005, **109**, 12124-12132.
105. U. Domańska, Z. Żołek-Tryznowska and M. Królikowski, *J. Chem. Eng. Data*, 2007, **52**, 1872-1880.
106. U. Domańska, P. Okuniewska and M. Krolikowski, *Fluid Phase Equilib.*, 2016, **423**, 109-119.
107. U. Domańska and L. M. Casas, *J. Phys. Chem. B*, 2007, **111**, 4109-4115.
108. U. Domańska, M. Wlazło, M. Karpińska and M. Zawadzki, *Fluid Phase Equilib.*, 2017, **449**, 1-9.
109. C. Jia, Y. Cao, T. Zuo, R. Hu, T. Yao and H. Song, *J. Chem. Eng. Data*, 2015, **60**, 999-1005.
110. G. Fischer, G. Holl, T. M. Klapötke and J. J. Weigand, *Thermochim. Acta*, 2005, **437**, 168-178.
111. U. Domańska, M. Zawadzki, M. M. Tshibangu, D. Ramjugernath and T. M. Letcher, *J. Chem. Thermodyn.*, 2010, **42**, 1180-1186.
112. U. Domańska and M. Zawadzki, *J. Chem. Thermodyn.*, 2011, **43**, 989-995.
113. U. Domańska, M. Zawadzki, M. M. Tshibangu, D. Ramjugernath and T. M. Letcher, *J. Phys. Chem. B*, 2011, **115**, 4003-4010.
114. U. Domańska, M. Zawadzki and M. Zwolińska, *J. Chem. Thermodyn.*, 2011, **43**, 775-781.
115. M. Zawadzki and U. Domańska, *J. Chem. Thermodyn.*, 2012, **48**, 276-283.
116. U. Domańska, M. Królikowski, M. Zawadzki and A. Wróblewska, *J. Chem. Thermodyn.*, 2016, **102**, 357-366.
117. Y. Funasako, K.-i. Abe and T. Mochida, *Thermochim. Acta*, 2012, **532**, 78-82.
118. Ł. Marcinkowski, A. Kloskowski and J. Namiesnik, *J. Chem. Eng. Data*, 2014, **59**, 585-591.
119. J. Vitorino, F. Agapito, M. F. M. Piedade, C. E. S. Bernardes, H. P. Diogo, J. P. Leal and M. E. M. da Piedade, *J. Chem. Thermodyn.*, 2014, **77**, 179-189.
120. X.-H. Sun, Y.-F. Liu, Z.-C. Tan, Y.-Y. Di, H.-F. Wang and M.-H. Wang, *J. Chem. Thermodyn.*, 2004, **36**, 895-899.
121. U. Domańska, A. Wiśniewska, Z. Dąbrowski and M. Więckowski, *J. Mol. Liq.*, 2018, **255**, 504-512.
122. F. J. M. Casado, A. S. Arenas, M. V. G. Pérez, M. I. R. Yélamos, S. L. de Andrés and J. A. R. Cheda, *J. Chem. Thermodyn.*, 2007, **39**, 455-461.
123. G. J. Janz, *Molten Salts Handbook*, Academic, 1967.
124. T. Endo, T. Morita and K. Nishikawa, *Chem. Phys. Lett.*, 2011, **517**, 162-165.
125. J. L. Aragones, E. Sanz, C. Valeriani and C. Vega, *J. Chem. Phys.*, 2012, **137**, 104507.
126. U. Domańska and A. Marciniak, *J. Chem. Eng. Data*, 2003, **48**, 451-456.
127. H. Sifaoui, A. Ait-Kaci, A. Modarressi and M. Rogalski, *Thermochim. Acta*, 2007, **456**, 114-119.
128. P. B. P. Serra, F. M. S. Ribeiro, M. A. A. Rocha, M. Fulem, K. Růžička, J. A. P. Coutinho and L. M. N. B. F. Santos, *J. Mol. Liq.*, 2017, **248**, 678-687.
129. J. Troncoso, C. A. Cerdeirin, Y. A. Sanmamed, L. Romani and L. P. N. Rebelo, *J. Chem. Eng. Data*, 2006, **51**, 1856-1859.
130. Z. H. Zhang, T. Cui, J. L. Zhang, H. Xiong, G. P. Li, L. X. Sun, F. Xu, Z. Cao, F.

Li and J. J. Zhao, *J. Therm. Anal. Calorim.*, 2010, **101**, 1143-1148.
131. M. W. Chase, *NIST-JANAF Thermochemical Tables, 4th Edition*, American Institute of Physics, 1998.
132. G. J. Janz and N. P. Bansal, *J. Phys. Chem. Ref. Data*, 1982, **11**, 505-693.
133. G. J. Janz, *J. Phys. Chem. Ref. Data*, 1988, **17**.
134. K. R. Seddon, A. Stark and M.-J. Torres, *ACS Symp. Ser.*, 2002, **819**.
135. T. Umecky, M. Kanakubo and Y. Ikushima, *Fluid Phase Equilib.*, 2005, **228-229**, 329-333.
136. K. R. Harris, L. A. Woolf and M. Kanakubo, *J. Chem. Eng. Data*, 2005, **50**, 1777-1782.
